# Integrative Genome-wide Association Meta-analysis of Aortic Aneurysm and Dissection Identifies Five Novel Genes

**DOI:** 10.1093/gpbjnl/qzaf039

**Published:** 2025-04-29

**Authors:** Yifan Du, Yunlong Guan, Zhonghe Shao, Minghui Jiang, Minghan Qu, Yifan Kong, Hongji Wu, Da Luo, Shu Peng, Si Li, Xi Cao, Jing Chen, Ping Ye, Jiahong Xia, Xingjie Hao

**Affiliations:** Department of Epidemiology and Biostatistics, Ministry of Education Key Laboratory of Environment and Health, School of Public Health, Tongji Medical College, Huazhong University of Science and Technology, Wuhan 430030, China; Department of Cardiovascular Surgery, Union Hospital, Tongji Medical College, Huazhong University of Science and Technology, Wuhan 430022, China; Department of Epidemiology and Biostatistics, Ministry of Education Key Laboratory of Environment and Health, School of Public Health, Tongji Medical College, Huazhong University of Science and Technology, Wuhan 430030, China; Department of Epidemiology and Biostatistics, Ministry of Education Key Laboratory of Environment and Health, School of Public Health, Tongji Medical College, Huazhong University of Science and Technology, Wuhan 430030, China; Department of Epidemiology and Biostatistics, Ministry of Education Key Laboratory of Environment and Health, School of Public Health, Tongji Medical College, Huazhong University of Science and Technology, Wuhan 430030, China; Department of Epidemiology and Biostatistics, Ministry of Education Key Laboratory of Environment and Health, School of Public Health, Tongji Medical College, Huazhong University of Science and Technology, Wuhan 430030, China; Department of Epidemiology and Biostatistics, Ministry of Education Key Laboratory of Environment and Health, School of Public Health, Tongji Medical College, Huazhong University of Science and Technology, Wuhan 430030, China; Department of Epidemiology and Biostatistics, Ministry of Education Key Laboratory of Environment and Health, School of Public Health, Tongji Medical College, Huazhong University of Science and Technology, Wuhan 430030, China; Department of Cardiology, Renmin Hospital of Wuhan University; Cardiovascular Research Institute, Wuhan University, Wuhan 430060, China; Department of Thoracic Surgery, Tongji Hospital, Tongji Medical College, Huazhong University of Science and Technology, Wuhan 430030, China; Department of Epidemiology and Biostatistics, Ministry of Education Key Laboratory of Environment and Health, School of Public Health, Tongji Medical College, Huazhong University of Science and Technology, Wuhan 430030, China; Department of Epidemiology and Biostatistics, Ministry of Education Key Laboratory of Environment and Health, School of Public Health, Tongji Medical College, Huazhong University of Science and Technology, Wuhan 430030, China; Department of Cardiology, Renmin Hospital of Wuhan University; Cardiovascular Research Institute, Wuhan University, Wuhan 430060, China; Department of Cardiology, Central Hospital of Wuhan, Tongji Medical College, Huazhong University of Science and Technology, Wuhan 430014, China; Department of Cardiovascular Surgery, Union Hospital, Tongji Medical College, Huazhong University of Science and Technology, Wuhan 430022, China; Department of Epidemiology and Biostatistics, Ministry of Education Key Laboratory of Environment and Health, School of Public Health, Tongji Medical College, Huazhong University of Science and Technology, Wuhan 430030, China

**Keywords:** Genome-wide association study, Aortic aneurysm, Aortic dissection, Susceptibility locus, Integrative study

## Abstract

Aortic aneurysm and dissection (AAD) is a multifaceted condition characterized by significant genetic predisposition and a considerable contribution to cardiovascular-related mortality. Previous studies have suggested that AAD subtypes share similar genetic mechanisms; however, these studies investigated the subtypes separately. Here, we performed a large genome-wide association study (GWAS) meta-analysis for AAD by combining its subtypes, including 11,148 cases and 708,468 controls of European ancestry. We identified 24 susceptibility loci, including four novel loci at 1p21.2 (*PALMD*), 2p22.2 (*CRIM1*), 6q22.1 (*FRK*), and 12q14.3 (*HMGA2*), which were partially validated in both internal and external populations. Cell type-specific analysis highlighted the artery as the most relevant tissue where the susceptibility variants may exert their effects in a tissue-specific manner. By using four approaches, we prioritized 53 genes, reinforcing the importance of elastic fiber formation and transforming growth factor-beta (TGF-β) signaling in the formation of AAD, and suggested potential target drugs for the treatment. Additionally, various cardiovascular diseases were genetically correlated with AAD, and several cardiovascular risk factors [*e.g.*, body mass index (BMI), lipid levels, and pulse pressure] showed causal associations with AAD, underscoring their shared genetic structures and mechanisms underlying the comorbidity. Moreover, five prioritized genes (*PALMD*, *CRIM1*, *FRK*, *HMGA2*, and *NT5DC1*) at the novel loci were supported as regulators of smooth muscle and endothelial cell functions through *ex vivo* and *in vitro* experiments. Together, these findings enhance our understanding of the genetic architecture of AAD and provide novel insights into future biological mechanism studies and therapeutic strategies.

## Graphical abstract

**Figure:**
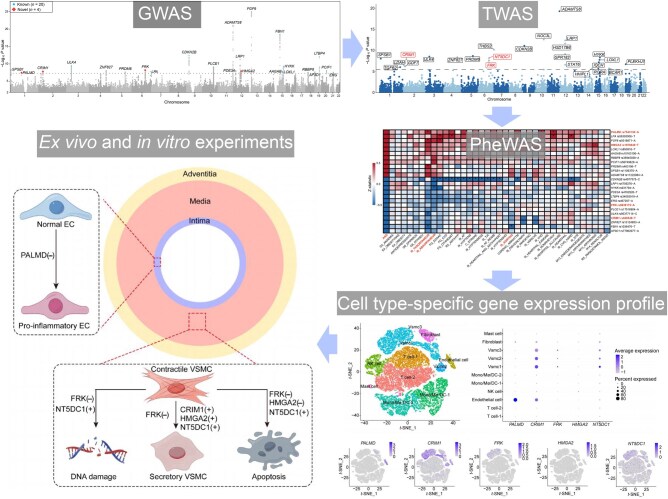

## Introduction

Aortic aneurysm and dissection (AAD) is a common and lethal disease with no effective cure. Based on the aortic region involved, AAD can be classified as either thoracic AAD (TAAD) or abdominal AAD (AAAD). AAD causes more than 10,000 deaths per year in the United States [1] and 150,000–200,000 deaths per year worldwide [2]. Although no medications are currently effective in preventing AAD progression, these deaths can be reduced by identifying high-risk individuals and applying appropriate management strategies to avoid aneurysm ruptures [3–6]. The development of AAD is influenced by multifactorial etiology, encompassing both genetic predispositions and environmental factors, including smoking, atherosclerosis, and hypertension [6–8]. A family history of AAD could also increase the individual’s risk of AAD [7,9]. The shared genetic determinants of AAD across subtypes remain largely unknown.

The origins of smooth muscle cells (SMCs) in the thoracic and abdominal aortas are different, resulting in distinct pathogenesis mechanisms of TAAD and AAAD [6]. Despite these differences, TAAD and AAAD share several common pathological features, including extracellular matrix (ECM) remodeling and SMC loss [7,10]. Additionally, the transforming growth factor-beta (TGF-β) signaling pathway and LRP1 pathway have been identified as potential biological pathways shared by both TAAD and AAAD [7,11]. Nevertheless, each subtype also demonstrates unique pathological characteristics. For instance, lipid metabolism and inflammatory responses are major contributors to AAAD pathogenesis but have minimal impact on TAAD [10–12]. Previous genome-wide association studies (GWASs) have been conducted for AAD mainly in populations of European ancestry; however, TAAD and AAAD were often analyzed separately [11–18]. The latest TAAD GWAS has identified 21 genes for TAAD, of which 7 genes (*TCF7L2*, *LRP1*, *SPSB1*, *ZNF827*, *PRDM6*, *PLCE1*, and *ADAMTS8*) are also associated with the genetic risk of AAAD [11]. Moreover, the largest AAAD GWAS has revealed that 24 AAAD index variants are also associated with TAAD, highlighting a moderate genetic correlation between these two subtypes [11].

To increase sample size and boost the power of GWASs, several studies have combined different histological subtypes. This strategy has enabled the identification of additional susceptibility loci and provided novel insights into the genetic architecture of complex diseases. For example, GWASs on inflammatory bowel disease often include two subtypes, Crohn’s disease and ulcerative colitis [19,20]; those on osteoarthritis and venous thromboembolism incorporate subtypes from different body sites [21–24]; GWAS on stroke includes subtypes of ischemic stroke and hemorrhagic stroke [25]; and pan-cancer GWASs encompass multiple cancer subtypes [26,27]. Furthermore, an increasing amount of tissue-specific and cell type-specific omics data, along with biobank-scale GWAS summary statistics, have become publicly accessible [28–33]. Moreover, lots of state-of-the-art computational approaches have been developed for post-GWAS integrative analyses [34,35]. Previous studies have performed post-GWAS analysis of cell type-specific enrichment and genetic correlation estimation for TAAD and AAAD separately. For instance, risk variants associated with AAAD are enriched in the functional regions of endothelial cells (ECs) and vascular SMCs of abdominal aortic tissue [11], whereas TAAD risk variants are enriched in vascular SMCs and fibroblasts of thoracic aortic tissue [12].

In our study, we performed a comprehensive GWAS for AAD by combining TAAD, AAAD, and aortic aneurysm of unspecified sites to uncover additional novel mechanistic candidate loci. We then integrated multiple omics datasets and phenotypic traits to identify relevant cell types, prioritized putative genes, and explored the genetic relationships between AAD and other traits. We further validated the prioritized genes at the novel loci by *ex vivo* and *in vitro* experiments.

## Results

### GWAS meta-analysis identifies four novel loci

To enhance the understanding of AAD’s genetic architecture, we performed a GWAS meta-analysis involving 11,148 cases and 708,468 controls, utilizing European-ancestry data from the UK Biobank (UKB) [32] and the FinnGen study [36] (Figure S1). Comprehensive details on GWAS quality control, sample size, and phenotype definitions are provided in the “Materials and methods” section. The liability heritability was 0.102 [standard error of the mean (SEM) = 0.010] when setting the population prevalence to the sample prevalence of 1.5%.

**Figure 1:**
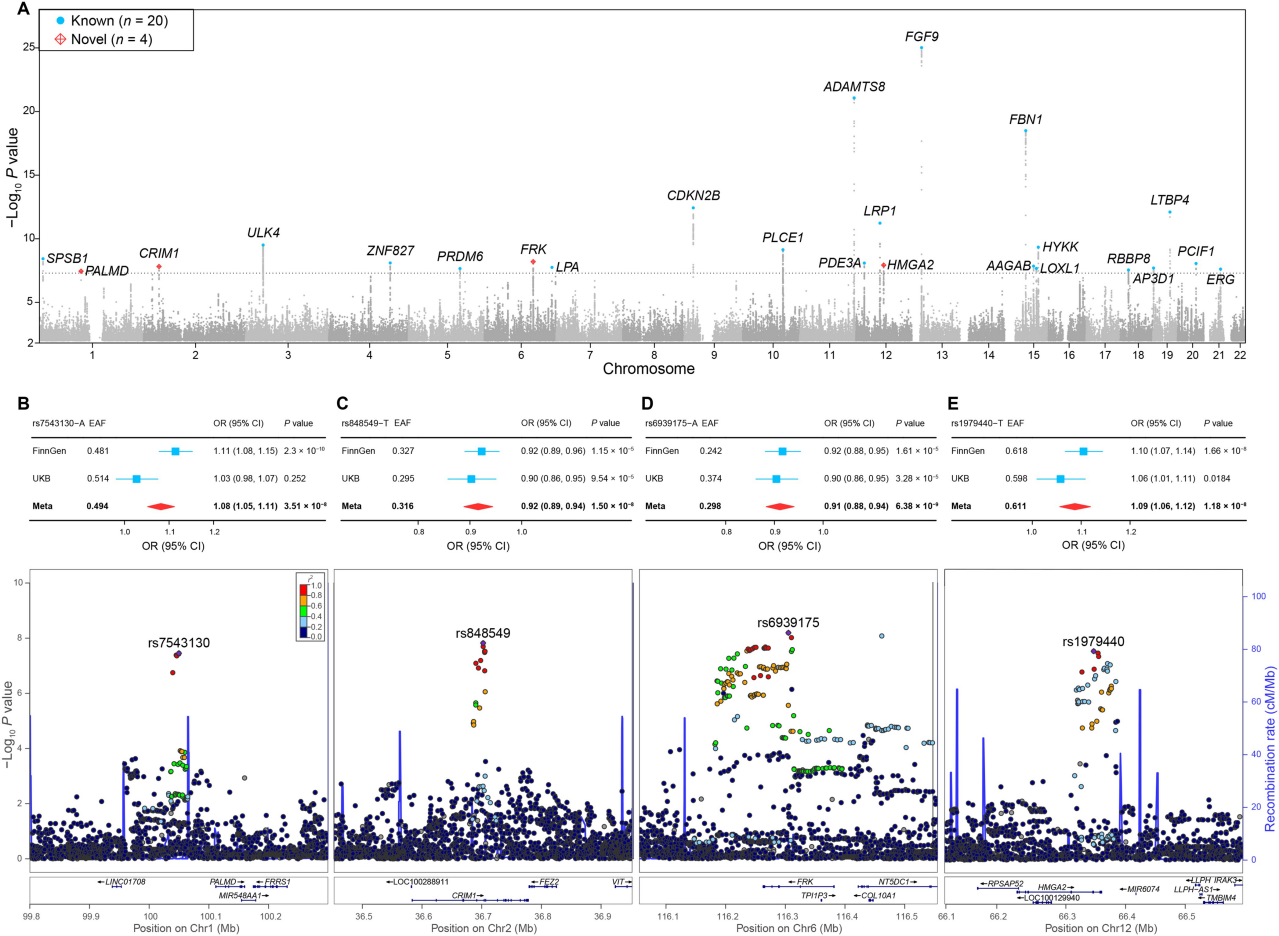
GWAS meta-analysis of AAD in 719,616 individuals **A**. Manhattan plot displaying the *P* values of variants. The gray dashed line marks the genome-wide significance threshold of 5 × 10^−8^. Top variants in the known loci are shown in blue, while those in novel loci are highlighted in red. **B**.–**E**. Forest plots and regional plots for the novel loci *PALMD* at 1p21.2 (B), *CRIM1* at 2p22.2 (C), *FRK* at 6q22.1 (D), and *HMGA2* at 12q14.3 (E). In the forest plots, blue square represents the effect estimation in each study, and red diamond represents the effect estimation in the meta-analysis. GWAS, genome-wide association study; OR, odds ratio; CI, confidence interval; Chr, chromosome; EAF, effect allele frequency; AAD, aortic aneurysm and dissection; UKB, UK Biobank.

By using the clumping approach and stepwise conditional analysis, we identified independent signals (*P* < 5 × 10^−8^) at 24 loci (**Figure 1**A; **Table 1**), including 20 previously identified loci for AAD, AAD histological subtypes of AAAD and TAAD, coronary artery aneurysm and dissection, and brain aneurysm. Four novel loci were *PALMD* at 1p21.2 (*P* = 3.51 × 10^−8^ for rs7543130), *CRIM1* at 2p22.2 (*P* = 1.5 × 10^−8^ for rs848549), *FRK* at 6q22.1 (*P* = 6.38 × 10^−9^ for rs6939175), and *HMGA2* at 12q14.3 (*P* = 1.8 × 10^−8^ for rs1979440) (Figure 1B–E). The internal validation study for the novel loci in UKB and the FinnGen study showed that all lead variants had consistent effect directions and met the nominal significance threshold (*P* < 0.05), with the exception of rs7543130 at *PALMD* (*P* = 0.252 in UKB) (Figure 1B–E). For external validation study, we evaluated these loci in the BioBank of Japan (BBJ) and Michigan Genomics Initiative (MGI). All lead variants exhibited consistent effect directions except for rs1979440 at *HMGA2* (File S1; Figure S2).

**Table 1:** Twenty-four loci identified in the genome-wide meta-analysis of AAD

Locus	SNP	Position	Gene	EA/NEA	EAF	UK Biobank		FinnGen study		Meta	
						OR (95% CI)	*P*	OR (95% CI)	*P*	OR (95% CI)	*P*
1	rs1106370	1:9443340	*SPSB1*	A/G	0.49	1.08 (1.03–1.13)	8.52 × 10^−4^	1.09 (1.06–1.13)	3.01 × 10^−7^	1.09 (1.06–1.12)	3.70 × 10^−9^
**2**	**rs7543130**	**1:100049785**	***PALMD***	**A/C**	**0.49**	**1.03 (0.98**–**1.07)**	**2.52 × 10^−1^**	**1.11 (1.08–1.15)**	**2.30 × 10^−10^**	**1.08 (1.05–1.11)**	**3.51 × 10^−8^**
**3**	**rs848549**	**2:36702389**	***CRIM1***	**T/C**	**0.32**	**0.9 (0.86–0.95)**	**9.54 × 10^−5^**	**0.92 (0.89–0.96)**	**1.15 × 10^−5^**	**0.92 (0.89–0.94)**	**1.50 × 10^−8^**
4	rs56377114	3:41869475	*ULK4*	C/G	0.81	0.89 (0.84–0.95)	2.00 × 10^−4^	0.89 (0.86–0.93)	9.23 × 10^−8^	0.89 (0.86–0.93)	3.07 × 10^−10^
5	rs13124853	4:146784774	*ZNF827*	A/G	0.59	0.92 (0.88–0.96)	4.64 × 10^−4^	0.92 (0.89–0.95)	1.40 × 10^−6^	0.92 (0.89–0.95)	7.98 × 10^−9^
6	rs463106	5:122502567	*PRDM6*	T/C	0.53	1.06 (1.01–1.11)	1.67 × 10^−2^	1.10 (1.06–1.14)	4.68 × 10^−8^	1.08 (1.05–1.11)	2.17 × 10^−8^
**7**	**rs6939175**	**6:116305183**	***FRK***	**A/G**	**0.30**	**0.9 (0.86–0.95)**	**3.28 × 10^−5^**	**0.92 (0.88–0.95)**	**1.61 × 10^−5^**	**0.91 (0.88–0.94)**	**6.38 × 10^−9^**
8	rs56393506	6:161089307	*LPA*	T/C	0.18	1.15 (1.08–1.22)	6.16 × 10^−6^	1.09 (1.04–1.14)	1.16 × 10^−4^	1.11 (1.07–1.15)	1.77 × 10^−8^
	rs140570886	6:161013013		T/C	0.99	0.74 (0.63–0.87)	3.05 × 10^−4^	0.72 (0.61–0.85)	8.43 × 10^−5^	0.73 (0.65–0.82)	2.19 × 10^−7^*
9	rs4977575	9:22124744	*CDKN2B*	C/G	0.55	0.89 (0.85–0.93)	2.80 × 10^−7^	0.91 (0.88–0.94)	3.83 × 10^−8^	0.90 (0.88–0.93)	3.75 × 10^−13^
10	rs17516904	10:96067242	*PLCE1*	A/G	0.16	0.89 (0.83–0.95)	4.76 × 10^−4^	0.88 (0.84–0.92)	9.50 × 10^−8^	0.89 (0.85–0.92)	7.28 × 10^−10^
	rs10736085	10:95893052		A/T	0.54	0.94 (0.9–0.98)	5.99 × 10^−3^	0.91 (0.88–0.94)	9.20 × 10^−8^	0.92 (0.90–0.95)	1.10 × 10^−8^*
11	rs11222084	11:130273230	*ADAMTS8*	A/T	0.65	1.18 (1.13–1.24)	4.23 × 10^−12^	1.14 (1.10–1.18)	9.75 × 10^−13^	1.16 (1.12–1.19)	8.55 × 10^−22^
12	rs4762926	12:20244126	*PDE3A*	T/G	0.25	0.91 (0.86–0.96)	2.00 × 10^−4^	0.91 (0.87–0.95)	3.44 × 10^−6^	0.91 (0.88–0.94)	8.11 × 10^−9^
13	rs4759276	12:57526646	*LRP1*	A/G	0.38	0.89 (0.85–0.94)	3.89 × 10^−6^	0.91 (0.88–0.94)	6.38 × 10^−8^	0.90 (0.88–0.93)	5.82 × 10^−12^
**14**	**rs1979440**	**12:66346624**	***HMGA2***	**T/C**	**0.61**	**1.06 (1.01–1.11)**	**1.84 × 10^−2^**	**1.10 (1.07–1.14)**	**1.66 × 10^−8^**	**1.09 (1.06–1.12)**	**1.18 × 10^−8^**
15	rs9316871	13:22861921	*FGF9*	A/G	0.79	1.22 (1.15–1.29)	1.84 × 10^−11^	1.2 (1.15–1.25)	1.93 × 10^−17^	1.21 (1.17–1.25)	9.57 × 10^−26^
16	rs1036476	15:48914775	*FBN1*	T/C	0.92	0.79 (0.74–0.85)	4.31 × 10^−11^	0.81 (0.76–0.86)	1.12 × 10^−10^	0.80 (0.76–0.84)	3.15 × 10^−19^
17	rs10163156	15:67503637	*AAGAB*	A/G	0.42	1.09 (1.04–1.14)	3.32 × 10^−4^	1.08 (1.05–1.12)	3.72 × 10^−6^	1.08 (1.05–1.12)	1.41 × 10^−8^
18	rs893816	15:74228464	*LOXL1*	T/C	0.33	1.07 (1.02–1.12)	6.91 × 10^−3^	1.10 (1.06–1.14)	1.63 × 10^−7^	1.09 (1.06–1.12)	2.15 × 10^−8^
19	rs931794	15:78826180	*HYKK*	A/G	0.66	0.92 (0.87–0.96)	2.99 × 10^−4^	0.91 (0.88–0.94)	9.26 × 10^−8^	0.91 (0.89–0.94)	4.62 × 10^−10^
20	rs35945038	18:20245248	*RBBP8*	A/G	0.32	1.10 (1.05–1.16)	5.23 × 10^−5^	1.08 (1.04–1.12)	4.02 × 10^−5^	1.09 (1.06–1.12)	2.83 × 10^−8^
21	rs77962077	19:2109480	*AP3D1*	A/G	0.07	0.87 (0.77–1.00)	4.38 × 10^−2^	0.83 (0.78–0.89)	3.33 × 10^−8^	0.84 (0.79–0.89)	2.01 × 10^−8^
22	rs34093919	19:41117300	*LTBP4*	A/G	0.02	0.62 (0.48–0.80)	2.22 × 10^−4^	0.64 (0.56–0.74)	1.15 × 10^−10^	0.64 (0.56–0.72)	7.96 × 10^−13^
23	rs58749629	20:44571317	*PCIF1*	A/G	0.14	1.18 (1.11–1.26)	5.04 × 10^−8^	1.08 (1.03–1.13)	1.64 × 10^−3^	1.12 (1.08–1.17)	8.80 × 10^−9^
24	rs467207	21:39996082	*ERG*	A/G	0.25	0.93 (0.88–0.99)	1.54 × 10^−2^	0.90 (0.87–0.93)	7.86 × 10^−8^	0.91 (0.88–0.94)	2.40 × 10^−8^

*Note*: The lead SNP with the lowest *P* value and any conditionally significant SNPs in each locus are presented. The gene corresponds to the nearest protein-coding gene to the lead SNP. *, the conditional *P* values are 3.25 × 10^−8^ for rs140570886 and 1.69 × 10^−11^ for rs10736085. The novel loci are shown in bold. SNP, single nucleotide polymorphism; EA, effect allele; NEA, non-effect allele; EAF, effect allele frequency; OR, odds ratio; CI, confidence interval; AAD, aortic aneurysm and dissection.

### Relevant tissues for AAD by partitioning the heritability

**Figure 2:**
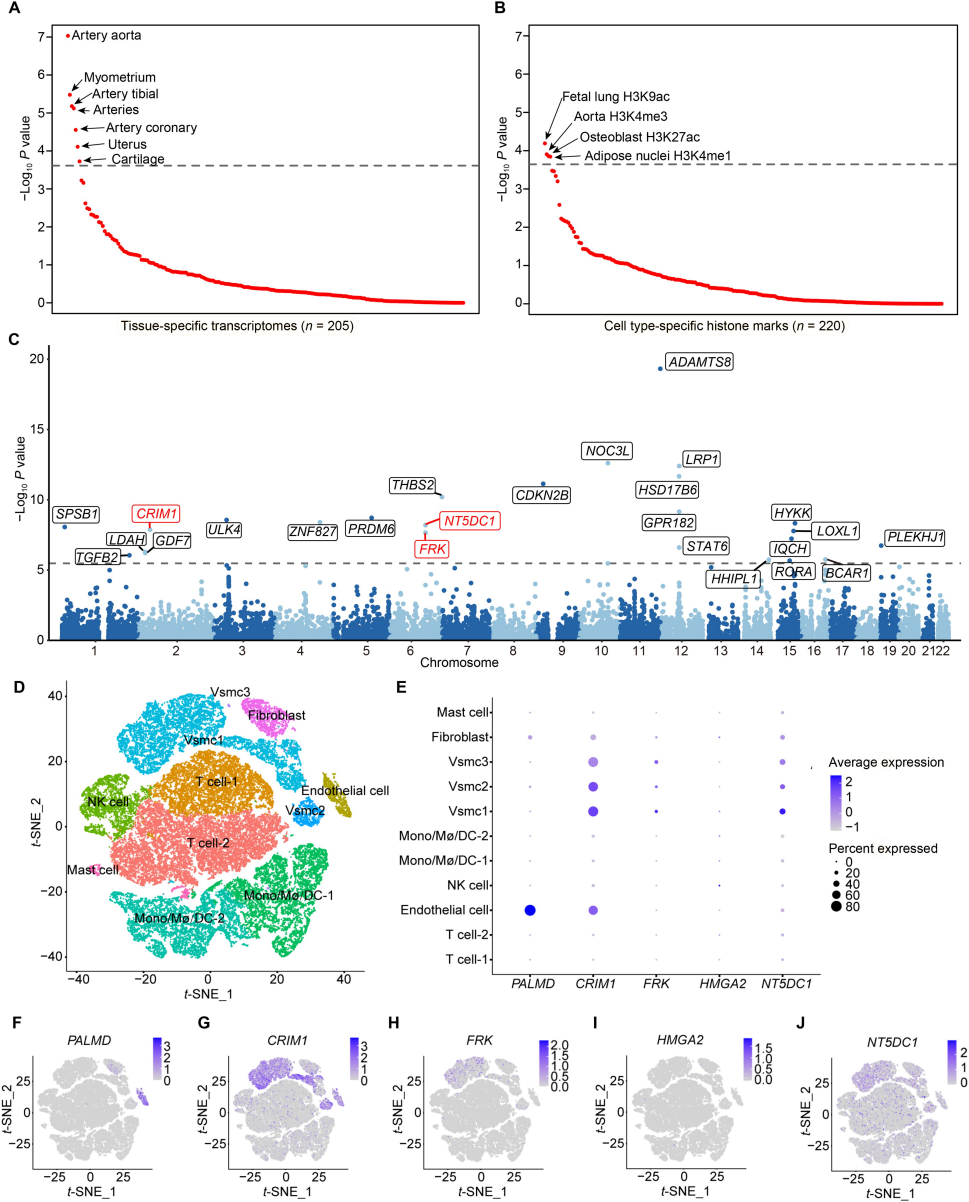
Relevant tissues and cell types for AAD **A**. Enrichment analysis of relevant tissues based on gene expression in 205 tissues from human, mouse, and rat. The dashed line indicates the Bonferroni-corrected significance level at 0.0002. **B**. Enrichment analysis of relevant cell types based on histone marks in 220 cell types from human. The dashed line indicates the Bonferroni-corrected significance level at 0.0002. **C**. Manhattan plot for the *P* values of genes in artery-specific TWAS. The dashed line indicates the transcriptome-wide significance level of 3.32 × 10^−6^. The genes in red mean those near the newly discovered loci. **D**. Cell type landscape in human sporadic type A aortic dissection based on scRNA-seq. **E**. Dot plot for five prioritized genes at the novel loci based on gene expression in different cell types. **F**.–**J**. Relative expression of *PALMD* (F), *CRIM1* (G), *FRK* (H), *HMGA2* (I), and *NT5DC1* (J) in different cell types. Vsmc, vascular smooth muscle cell; Mono/Mϕ/DC, monocyte/macrophage/dendritic cell; NK, natural killer; scRNA-seq, single-cell RNA sequencing; TWAS, transcriptome-wide association study; *t*-SNE, *t*-distributed stochastic neighbor embedding.

We used stratified linkage disequilibrium (LD) score regression (LDSC) to pinpoint the relevant tissues or cell types based on their specific gene expression and histone modification marks. The specific gene expression profiles from the aorta artery showed the most significant enrichment for the GWAS risk variants (*P* = 9.29 × 10^−8^) (**Figure 2**A). Other arteries, including the tibial and coronary arteries, also exhibited significant enrichment (*P* < 0.0002). Interestingly, we observed enrichment signals in the myometrium, uterus, and cartilage. In addition, the specific histone marks in four cell types (lung, aorta artery, osteoblast, and adipose nuclei) were significantly associated with AAD (*P* < 0.0002) (Figure 2B). Actually, osteoblasts are nearly indistinguishable from fibroblasts during cell culture [37]. The main components of myometrium, uterus, cartilage, and lung are SMCs or fibroblasts, both of which play critical roles in AAD development. Adipose can also contribute to the progression of AAD.

### Gene prioritization for AAD

For the identification of AAD candidate genes, we employed four approaches and prioritized 53 distinct genes, including 24 genes based on their proximity to the lead variants (Table 1), 7 genes identified through significant nonsynonymous variants (Table S1), 29 genes from artery-specific expression quantitative trait locus (eQTL) mapping (Table S2), and 25 genes from artery-specific transcriptome-wide association study (TWAS) (Table S3). Among these genes, *ULK4*, *ADAMTS8*, and *LOXL1* were identified by four approaches, and *CRIM1*, *ZNF827*, *PRDM6*, *FRK*, *LRP1*, and *NOC3L* were prioritized by three approaches (Table S4). Although the artery-specific TWAS prioritized seven genes (*TGFB2*, *GDF7*, *LDAH*, *THBS2*, *HHIPL1*, *RORA*, and *BCAR1*) outside the susceptibility loci identified in our GWAS-meta analysis (Figure 2C), these genes were located at the known loci for AAD, AAAD, or brain aneurysm in the previous studies [11,16,17,38,39].

At the novel loci, both the artery-specific TWAS and eQTL mapping approaches prioritized another gene, *NT5DC1* at 6q22.1, where the gene nearest to the lead variant is *FRK*. At the novel locus 2p22.2, *CRIM1* was the unique prioritized gene by approaches of position mapping, artery-specific TWAS, and eQTL mapping. Notably, the GWAS signals and eQTLs for *CRIM1*, *FRK*, and *NT5DC1* were colocalized with a posterior probability of colocalization *H*_4_ > 0.8 (Table S3). In addition, *PALMD* and *HMGA2* were unique genes only prioritized by position mapping at 1p21.2 and 12q14.3, respectively. To sum up, five genes (*PALMD*, *CRIM1*, *FRK*, *HMGA2*, and *NT5DC1*) were prioritized at these four novel loci. These genes were moderately or highly expressed in bulk tissues, such as artery, fibroblast, and adipose (Figure S3). In the high-resolution cell types identified by single-cell RNA sequencing (scRNA-seq) analysis of thoracic aorta specimens, *PALMD* was highly expressed in the ECs; *CRIM1* and *NT5DC1* were highly expressed in the SMCs; *FRK* was moderately expressed in SMCs (Figure 2D–J). Meanwhile, in human abdominal aorta specimens (GSE224587), we observed similar expression patterns for these novel genes (Figure S4). Additionally, the functional annotations and drug targets for these prioritized genes are detailed in File S1, Figure S5, and Tables S5 and S6.

### Functional characterization of five genes at the novel loci

**Figure 3:**
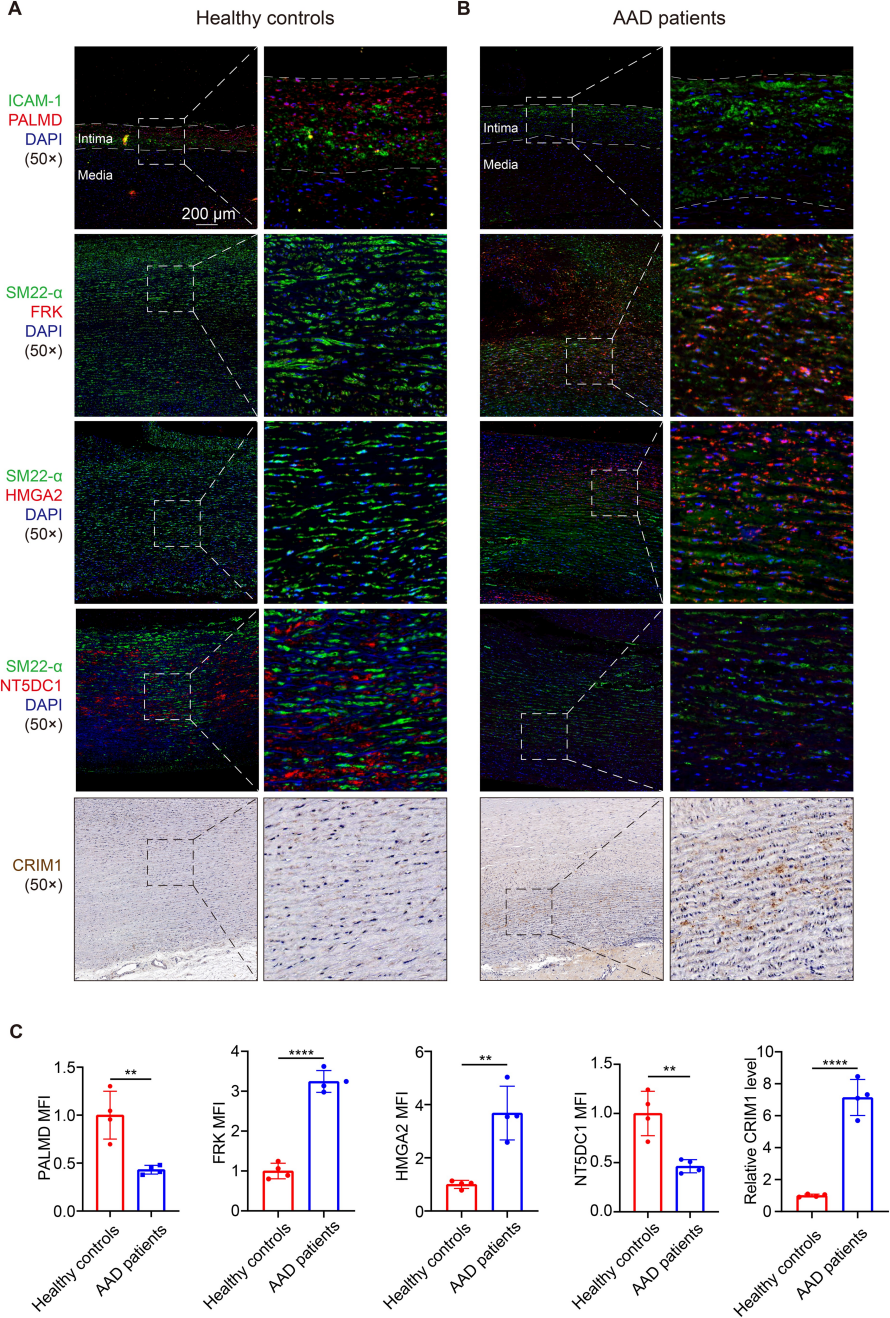
Localization and expression of prioritized genes in the aorta Representative images of aortic sections from healthy controls (**A**) and AAD patients (**B**) showing the localization of PALMD, FRK, HMGA2, NT5DC1, and CRIM1. Scale bar, 200 μm. **C**. Quantitative analysis of protein levels in healthy controls and AAD patients. Data are represented as mean ± SE (*n* = 4). Statistical significance was determined by Student’s *t*-test (**, *P* < 0.01; ****, *P* < 0.0001). MFI, mean fluorescence intensity; DAPI, 4′,6-diamidino-2-phenylindole; SE, standard error.

We evaluated the protein localization and expression of five prioritized genes at the novel loci in the aorta of AAD patients and healthy controls. PALMD was predominantly expressed in the intima of healthy controls. FRK, HMGA2, and CRIM1 were highly expressed in the SMCs of AAD patients, whereas NT5DC1 was highly expressed in the SMCs of healthy controls (**Figure 3**).

**Figure 4:**
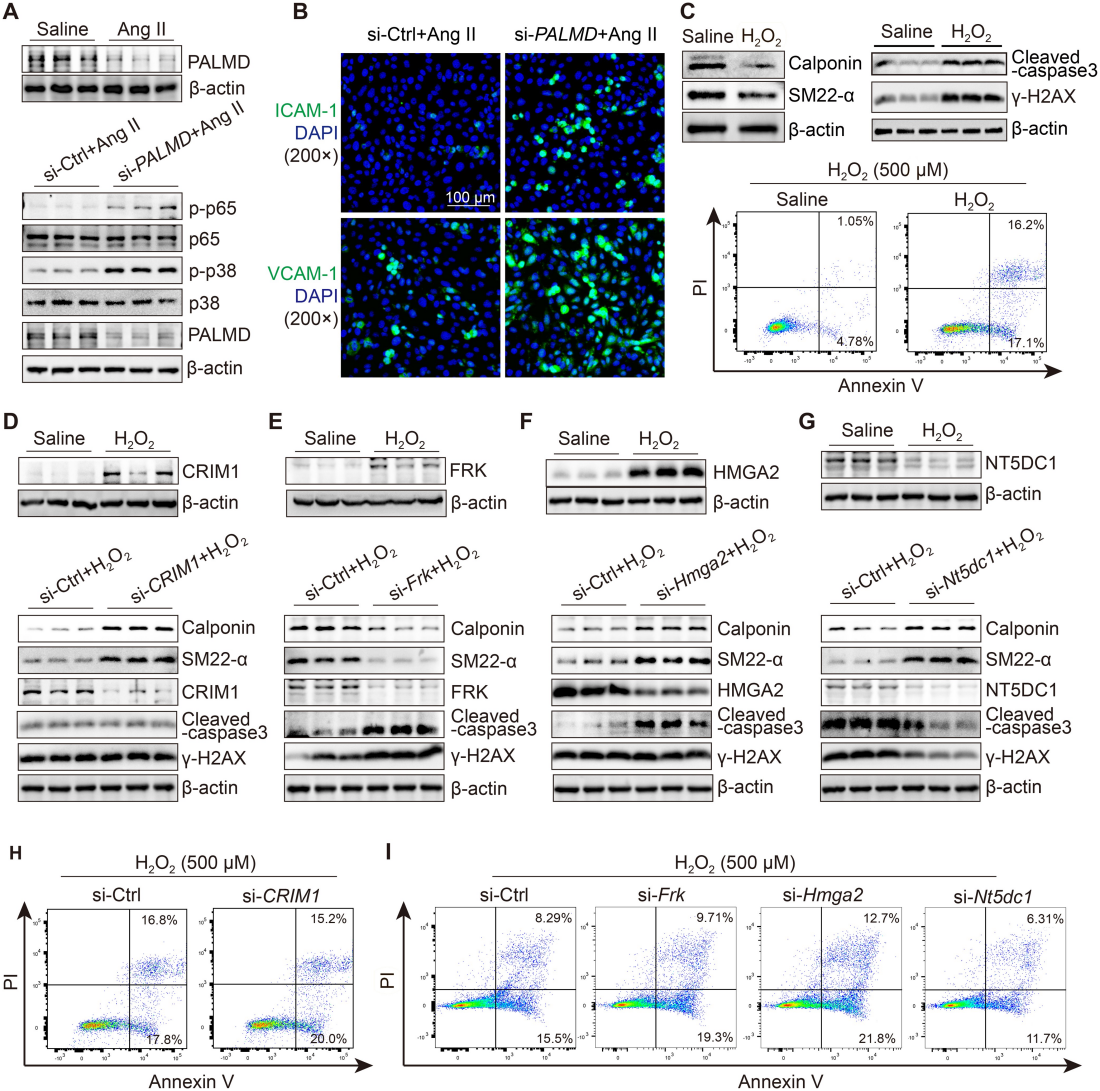
Functional assays of prioritized genes in vascular ECs and SMCs **A**. PALMD decreased after Ang II administration (top), and knockdown of *PALMD* activated p65 and p38 MAPK (bottom). **B**. Immunofluorescence staining showed that *PALMD* knockdown facilitated the expression of ICAM-1 and VCAM-1 in Ang II-challenged vascular ECs. **C**.–**G**. Protein level changes of SM22-α, calponin, cleaved-caspase3, γ-H2AX, CRIM1, FRK, HMGA2, and NT5DC1 in H_2_O_2_-challenged SMCs (C), as well as in H_2_O_2_-challenged SMCs with knockdown of *CRIM1* (D), *Frk* (E), *Hmga2* (F), and *Nt5dc1* (G). **H**. and **I**. Apoptotic proportions of H_2_O_2_-challenged SMCs with or without gene knockdown detected using flow cytometry. si-Ctrl refers to the negative control siRNA. siRNA, small interfering RNA; EC, endothelial cell; SMC, smooth muscle cell; Ang II, angiotensin II; p-p65, phosphorylated p65; p-p38, phosphorylated p38; PI, propidium iodide.

Human umbilical vein endothelial cells (HUVECs) were transfected with a small interfering RNA (siRNA) targeting *PALMD* or a negative control siRNA (**Figure 4**A). Endothelial dysfunction initiates vascular remodeling and inflammation, attracting immune cells to the medial layer, which is associated with the p38 and p65 signaling pathways and endothelial adhesion molecules (VCAM-1 and ICAM-1) [40]. Activation of p38 MAPK and p65 NF-κB leads to increased downstream inflammatory cytokine synthesis and plays a critical role in AAD formation [41,42]. *PALMD* knockdown in HUVECs exacerbated angiotensin II (Ang II)-induced increases in phosphorylated p65 (p-p65) and phosphorylated p38 (p-p38) levels (Figure 4A). Since downregulation of endothelial adhesion molecules (VCAM-1 and ICAM-1) has been found to attenuate Ang II-induced AAD progression [40,43], we also assessed their expression. Immunofluorescence staining revealed elevated levels of ICAM-1 and VCAM-1 in Ang II-challenged HUVECs with *PALMD* knockdown (Figure 4B, Figure S6).

Phenotypic switching and loss are significant characteristics of SMCs in AAD. Additionally, DNA damage is also commonly observed in AAD. In our *in vitro* experiments, we transfected human/mouse aortic SMCs with siRNAs targeting *CRIM1*, *Frk*, *Hmga2*, or *Nt5dc1*, followed by H_2_O_2_ challenge, and assessed the outcomes using Western blot (WB; for SM22-α, calponin, γ-H2AX, cleaved-caspase-3) and flow cytometry (FC). The results showed that contractile markers (SM22-α and calponin) were downregulated in challenged SMCs with *Frk* knockdown, but upregulated in challenged SMCs with *Hmga2*, *Nt5dc1*, or *CRIM1* knockdown. γ-H2AX levels were increased in challenged SMCs with *Frk* knockdown, but decreased with *Nt5dc1* knockdown. No remarkable change of γ-H2AX occurred in challenged SMCs with *CRIM1* or *Hmga2* knockdown. Moreover, apoptosis in H_2_O_2_-challenged SMCs was aggravated by *Frk* or *Hmga2* knockdown, alleviated by *Nt5dc1* knockdown, and unaffected by *CRIM1* silencing (Figure 4C–I, Figure S6).

### Genetic correlations with other diseases

**Figure 5:**
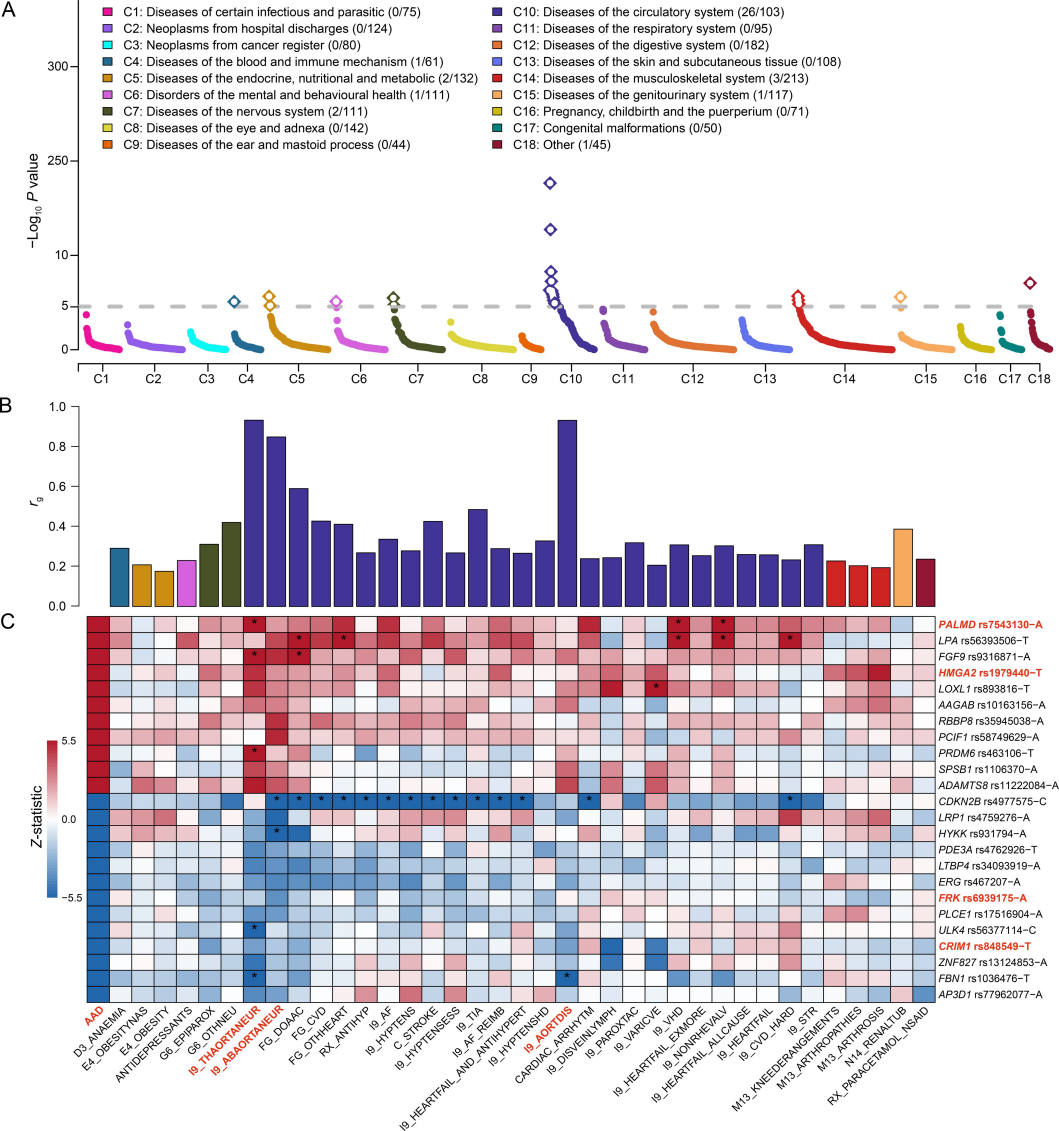
Phenome-wide genetic correlation analysis of AAD **A**. *P* values of genetic correlations between AAD and 1864 diseases in the FinnGen study. The gray dashed line indicates the significance threshold of 2.68 × 10^−5^ after Bonferroni correction for 1864 diseases. The number of diseases with significant genetic correlations and the total number of diseases in each disease category are provided in parentheses. **B**. Bar plot showing the significant genetic correlations (*r*_g_) of AAD with 37 diseases. Bars are color-coded by disease categories shown in (A). **C**. Heatmap illustrating the associations between AAD lead variants and the genetically correlated diseases. The strength and direction of associations are depicted using color-coded cells: red indicates positive effects on disease risk, while blue represents inverse associations. To enhance visualization, *Z*-statistics are truncated at ±5.5 (approximately *P* = 5 × 10^−8^). The AAD lead variants at the four novel loci, as well as AA histological subtypes, are labeled in red. The star indicates that the lead variant is genome-wide associated with the genetically correlated disease. AA, aortic aneurysm.

Phenome-wide genetic correlations were assessed to investigate the genetic overlap and similarities between AAD and 1864 diseases in the FinnGen study. After Bonferroni correction (*P* < 2.68 × 10^−5^), ADD showed significant genetic correlations with 37 diseases, ranging from 0.18 to 0.93 (**Figure 5**A and B; Table S7). Multiple cardiovascular diseases exhibited significant genetic correlations with AAD, and several cardiovascular risk factors [*e.g.*, body mass index (BMI), lipid levels, and pulse pressure] showed causal associations with AAD (File S1; Figures S7 and S8). AAD showed strong genetic correlations with its histological subtypes: thoracic aortic aneurysm (I9_THAORTANEUR, *r*_g_ = 0.93), dissection of aorta (I9_AORTDIS, *r*_g_ = 0.93), and abdominal aortic aneurysm (I9_ABAORTANEUR, *r*_g_ = 0.85).

The lead variants associated with AAD exhibited consistent effect directions at 635 loci (71.5%) across the genetically correlated diseases (Figure 5C; Table S8). For the four novel loci, the effect directions of the lead variants in *PALMD*, *HMGA2*, *FRK*, and *CRIM1* for AAD subtypes (*i.e.*, TAAD, AAAD, and dissection of aorta) were “+++”, “−−−”, “−−+”, and “+++”, respectively. In addition, in 57 of the 60 sets of results (95%), the lead variants for 20 previously reported loci showed consistent effect directions across three AAD subtypes, except for rs58749629 at *PCIF1* and rs4977575 at *CDKN2B* in TAAD, as well as rs35945038 at *RBBP8* in dissection of aorta. The lead variant rs7543130 at the novel locus *PALMD* was genome-wide significantly associated with thoracic aortic aneurysm (I9_THAORTANEUR, *P* = 2 × 10^−10^), valvular heart disease excluding rheumatic fever (I9_VHD, *P* = 1 × 10^−20^), and non-rheumatic valve diseases (I9_NONRHEVALV, *P* = 8.20 × 10^−21^) in the FinnGen study. Moreover, the lead variant rs4977575 at *CDKN2B* showed the greatest number (*n* = 14) of genome-wide significant associations with these genetically correlated diseases (Figure 5C).

## Discussion

We performed a comprehensive GWAS meta-analysis for AAD and identified 24 susceptibility loci, including four novel ones. For the four novel loci, rs7543130 is an intergenic variant located near the *PALMD* gene, whereas rs848549, rs6939175, and rs1979440 are intronic variants in *CRIM1*, *FRK*, and *HMGA2*, respectively. Cell type-specific analysis highlighted artery as the most relevant tissue where AAD-associated genes likely exert their effects. Our integrative analysis prioritized 53 genes, including five genes located at the novel loci. In addition, various cardiovascular diseases were genetically correlated with AAD, and several cardiovascular risk factors showed causal associations with AAD.

Our discovery GWAS meta-analysis included two large populations of European ancestry from UKB and the FinnGen study. Different from the previous GWASs that separately studied TAAD and AAAD [11–18], we combined all histological subtypes of AAD as cases, a strategy that has been proven effective in identifying more loci and generating novel insights into other diseases [19–27]. Most of the lead variants within the identified susceptibility loci exhibited consistent effect directions across cardiovascular diseases, particularly among AAD histological subtypes, suggesting shared mechanisms. We validated the novel loci in both internal and external populations, with the primary focus on the effect directions of the lead variants. The signals at two novel loci, *PALMD* and *CRIM1*, were relatively robust in the internal and external populations, and consistent across AAD histological subtypes. However, in the meta-analysis of AAD susceptibility loci based on the external populations from BBJ and MGI, the lead variant rs1979440 at the novel loci *HMGA2* was the unique variant showing an opposite effect direction. Similarly, variants at *FRK* displayed consistent effects in thoracic and abdominal aortic aneurysms, but exhibited an opposite effect direction in aortic dissection. In summary, our GWAS meta-analysis identified four novel loci that were partially validated across populations and correlated diseases, though further replication in larger and more diverse populations is required.

As expected, AAD signals exhibited enrichment in functional regions and genes highly expressed in arteries, as well as in tissues abundant with SMCs, fibroblasts, or adipose tissue — all of which are relevant to the histological subtypes of AAD, including TAAD and AAAD [11,12]. Based on position mapping and tissue-specific annotation, five genes (*PALMD*, *CRIM1*, *FRK*, *HMGA2*, and *NT5DC1*) were prioritized at the novel loci. *PALMD* is highly expressed in the ECs of the cardiovascular system [44]. *PALMD* deficiency may promote the development of calcific aortic valve disease by activating NF-κB signaling in valvular ECs [45]. Additionally, *PALMD* variants have been associated with aortic root size and aortic valve stenosis [46–48]. We showed that the knockdown of *PALMD* resulted in significantly elevated pro-inflammatory signals in ECs. Based on these findings, we speculate that PALMD plays a crucial role in maintaining EC homeostasis and may be involved in AAD pathogenesis. *CRIM1* encodes a transmembrane protein that anchors growth factors, such as vascular endothelial growth factor A, to the cell surface [49]. Furthermore, CRIM1 is involved in maintaining endothelial integrity and has a critical role in cardiovascular system development. *Crim1* deficiency causes primary defects in the extraglomerular vascular system in mice [50]. We demonstrated that knockdown of *CRIM1* in SMCs could increase the expression of SM22-α and calponin, promoting the contractile phenotype of SMCs, which could protect against the formation of AAD. However, *CRIM1* appears to have opposing effects in SMCs and ECs, suggesting a complex role in AAD progression. Further *in vivo* studies are necessary to determine its overall effect. *FRK* encodes a member of the SRC family of kinases that stabilizes PTEN and BRCA1 via tyrosine phosphorylation [51]. Additionally, it has been shown to inhibit the proliferation of human glioblastoma cells by reducing cyclin D1 nuclear accumulation [52]. Therefore, *FRK* may also regulate arterial SMCs proliferation. The variants in *FRK* are associated with apolipoprotein B and C-reactive protein, both of which are linked to coronary heart disease [53,54]. Our study found that *Frk* knockdown inhibited the contractile phenotype of SMCs and activated SMC apoptosis and DNA damage. Thus, *FRK* may act as a protective factor against aortic degeneration. *HMGA2* is an architectural transcription factor involved in DNA damage repair and stem cell self-renewal [55,56], potentially mitigating SMC loss and associated local inflammation — both essential for stabilizing aortic structure. The variants in *HMGA2* also showed associations with aortic root size [57] and cardiovascular risk factors of blood pressure and BMI [58,59]. Our experiment indicated that the knockdown of *Hmga2* could simultaneously promote SMC contractile phenotype and apoptosis, indicating its complex role in AAD pathophysiology. The function of *NT5DC1* is less well studied. It has been implicated in susceptibility to chronic obstructive pulmonary disease and as a marker for pancreatic adenocarcinoma [60,61]. In our study, *Nt5dc1* knockdown in SMCs significantly increased SM22-α and calponin expression, while downregulating proteins associated with DNA damage and apoptosis, suggesting that *NT5DC1* may aggravate AAD formation through its effects on SMCs.

In the phenome-wide genetic correlation analysis, AAD demonstrated significant positive genetic correlations with 37 other diseases, predominantly cardiovascular diseases. The moderate-to-strong genetic correlations with cardiovascular diseases emphasize their shared genetic architecture and mechanisms underlying comorbidities. Traditional cardiovascular risk factors, including low-density lipoprotein (LDL), total cholesterol (TC), triglyceride (TG), high-density lipoprotein (HDL), pulse pressure (PP), and BMI, were found to be causally associated with AAD. In any case, as the first study to comprehensively investigate the causal cardiovascular risk factors for AAD, we have identified several causal exposures that may either serve as common risk factors for various AAD subtypes or act as the predominant risk factors for specific AAD subtypes.

Several limitations warrant mention. First, although we tried to validate the novel loci in the internal and external populations, as well as AAD histological subtypes, we mainly focused on the sign of effect allele in the replication studies. These novel loci could not all be fully validated under a strict Bonferroni-corrected *P* value threshold. To provide more evidence to support the novel loci, we prioritized five genes by multi-omics integrative study and further conducted *ex vivo* and *in vitro* experiments to explore their potential genetic mechanisms at the novel loci. Second, while we proposed a list of potential drug targets for AAD treatment, we did not perform drug intervention experiments to validate the effects of these potential drugs due to limited experimental technology. As a proxy approach, we performed drug–target Mendelian randomization (MR) analysis, and successfully highlighted the significant potential of LPA as a treatment target for AAD. However, we failed to identify instrumental variables for other proteins, which prevented us from evaluating their potential as AAD targets. Third, we found that several common cardiovascular risk factors, namely BMI, lipids, and pulse pressure, showed causal associations with AAD. However, these cardiovascular risk factors are correlated, and their causal relationships may be mediated. Conducting mediation analyses across these risk factors and multiple AAD subtypes will comprehensively clarify the regulatory network.

In conclusion, this large-scale integrative genome-wide analysis of various AAD subtypes has provided novel insights into the genetic architecture and mechanism for AAD. Our study has developed a comprehensive atlas of candidate genes, relevant tissues and cell types, and pathways implicated in AAD formation. Furthermore, we have identified a list of potential drug targets for AAD and investigated its associations with other diseases and traits.

## Materials and methods

### UKB genotyping and quality control

The UKB cohort is a large population-based cohort comprising approximately 500,000 individuals with extensive phenotypic and genetic data. Detailed protocols of sample processing, genotyping, and quality control have been described elsewhere [32]. In this study, we used the imputed genotypes of 15,456,785 variants in 378,474 European-ancestry individuals, obtained from our previous work [62]. The UKB Application No. for this study is 88159.

### GWAS on AAD in UKB

The AAD phenotype in UKB was determined based on the first occurrence of any code mapped to the 3-character International Classification of Diseases 10th revision (ICD-10; category ID 1712 in UKB), which was achieved by integrating death register records, primary care data, hospital inpatient records, and self-reported medical conditions collected during the baseline assessments or subsequent visits to the UKB assessment center. AAD cases were defined as individuals diagnosed with AAD, corresponding to ICD-10 code I71, and the remaining participants were controls after further removing those with several circulatory system diseases [*i.e.*, atherosclerosis (I70), other aneurysm (I72), other peripheral vascular diseases (I73), arterial embolism and thrombosis (I74), other disorders of arteries and arterioles (I77), diseases of capillaries and disorders of arteries (I78), and arterioles and capillaries in diseases classified elsewhere (I79)], following the approach used in the FinnGen study. After quality control, as of July 2022, 3753 UKB participants of European ancestry were classified as cases, while 358,929 individuals of the same ancestry served as controls. Logistic regression implemented in PLINK [63] was applied to detect the AAD-associated variants with adjustments for sex, array batch, and the top 10 principal components (PCs) of genetic ancestry.

### GWAS summary statistics for AAD from the FinnGen study

Summary statistics for AAD in the FinnGen study (data freeze 9, April 2022, code I9_AORTANEUR) are publicly available at https://r9.finngen.fi/. In the FinnGen study, AAD cases were defined by ICD-10 code I71, with the exclusion of dissection of unspecified site of aorta (I71.0), resulting in 7395 cases. The remaining samples were controls (*n* = 349,539) after further removing those with atherosclerosis (I70), other aneurysm (I72), other peripheral vascular diseases (I73), arterial embolism and thrombosis (I74), other disorders of arteries and arterioles (I77), diseases of capillaries and disorders of arteries (I78), and arterioles and capillaries in diseases classified elsewhere (I79). The GWASs in the FinnGen study were conducted using Regenie [64], adjusting for sex, age, the top 10 PCs, and genotyping batch.

### GWAS meta-analysis

The inverse-variance weighted meta-analysis using the fixed effect model in METAL [65] was performed, with genomic control correction applied to each input study. A total of 9,797,427 variants common to both UKB and FinnGen were included for the subsequent analysis. Variants reaching genome-wide significance (*P* < 5 × 10^−8^) were identified. To define the independent loci for the associated variants, we first performed clumping to select the independent lead variant at each locus by setting LD *r*^2^ less than 0.1 within a 10-Mb region in PLINK [63]. The LD was calculated based on 1000 randomly selected European samples from UKB. Second, we merged adjacent lead variants that were located within 1000 kb into one locus. A total of 24 independent loci were identified, and the corresponding nearest genes were mapped. Furthermore, stepwise conditional analysis was conducted with genome-wide complex trait analysis (GCTA) [66] based on the aforementioned LD reference panel to detect additional significant signals within a locus.

Previously reported loci of AAAD, TAAD, coronary artery aneurysm and dissection, and brain aneurysm in the GWAS Catalog (as of September 2023) [33] and the latest studies [11,12,67] were downloaded. Loci that were significant in our GWAS meta-analysis were designated as novel only if they met both of the following criteria: (1) they did not pass the genome-wide significance threshold in the GWAS Catalog and latest publications, and (2) their lead variant was located over 1000 kb away from any previously reported index variant in the GWAS Catalog and latest publications.

### Tissue and cell type identification

LDSC [68] was used to assess whether AAD heritability was enriched in tissue-specific or cell type-specific functional annotations. These included 220 cell type-specific genomic functional regions defined by histone marks [69] and 205 tissue-specific gene expression sets [68]. A total of 53 non-tissue-specific baseline annotations [*e.g*., coding, 3′ untranslated region (UTR), 5′ UTR, promoter, and intron] [68,69] were included in the LDSC model to minimize confounding factors and enhance cell type-specific signals. LD scores were calculated based on the genotypes of Europeans from the 1000 Genomes Project [69] and downloaded from https://alkesgroup.broadinstitute.org/LDSCORE/. For cell type-specific and subsequent phenome-wide genetic correlation analyses, only HapMap 3 single nucleotide polymorphisms (SNPs) were used, with SNPs in the major histocompatibility complex (MHC) region excluded. Bonferroni correction was applied to account for multiple testing.

### Candidate gene mapping and prioritization

Four approaches were used to map variants to protein-coding genes, including position mapping by the lead variant, nonsynonymous variant mapping, artery-specific eQTL mapping based on Functional Mapping and Annotation (FUMA; https://fuma.ctglab.nl) [70], and artery-specific TWAS and colocalization analysis with the functional summary-based imputation (FUSION) pipeline [71] (more details in File S1). Previous studies reported that the heritability of TAAD and AAAD was mainly enriched in aortic tissue, and accordingly performed TWAS and eQTL mapping on TAAD and AAAD using aortic data, respectively [11,12]. To identify more candidate genes, we conducted eQTL mapping and TWAS using data from aorta artery, coronary artery, and tibial artery from the Genotype-Tissue Expression (GTEx) project (v8), respectively. The results from these three arteries were then combined to boost the statistical power.

### Cell type prioritization for genes at novel loci based on scRNA-seq data

To pinpoint cell types where the five prioritized genes (*PALMD*, *CRIM1*, *FRK*, *HMGA2*, and *NT5DC1*) at the novel loci might influence AAD risk, we utilized scRNA-seq datasets of thoracic aorta specimens (GSE155468) and abdominal aorta specimens (GSE224587) from TAAD/AAAD cases and controls. We reanalyzed these scRNA-seq datasets using Seurat (v4) [72]. Briefly, dimensionality reduction was conducted using *t*-distributed stochastic neighbor embedding (*t*-SNE), and cluster-defining genes were identified with the FindAllMarkers function. Feature plots, dot plots, and heatmaps were generated via Seurat (v4) [72].

### Immunofluorescence and immunohistochemistry analysis of aorta arteries

We obtained freshly isolated aortic tissues from the consenting heart transplant recipients or AAD patients. The aorta samples were fixed in 4% paraformaldehyde and paraffin-embedded. Sections were cut, dewaxed, antigen-retrieved, blocked with donkey serum, and incubated overnight with primary antibodies. The slides were washed with phosphate-buffered buffered saline with Tween 20 (PBST) and incubated with suitable secondary antibodies. Antibodies used are listed in File S1.

### EC inflammation assay for *PALMD*

Confluent HUVECs were transfected with si-*PALMD* at a final concentration of 37.5 nM for 24 h. Meanwhile, the control group was transfected with the negative control siRNA. The medium was then changed, and HUVECs were stimulated with Ang II (1 μM) for 24 h. After treatments, the protein levels of p-p65, p-p38, and PALMD were detected by WB and immunofluorescence staining. Detailed siRNA sequences are provided in Table S9, and antibodies used are listed in File S1.

### SMC functional assays for *CRIM1*, *Frk*, *Hmga2*, and *Nt5dc1*

Human aortic SMCs were transfected with si-*CRIM1*, while mouse aortic SMCs were transfected with si-*Frk*, si-*Hmga2*, or si-*Nt5dc1*, at a final concentration of 37.5 nM for 24 h. Meanwhile, the control group was transfected with the negative control siRNA. The medium was then changed, and SMCs were stimulated with H_2_O_2_ (500 μM) for 24 h. After treatments, the protein levels of SM22-α, calponin, cleaved-casepase3, γ-H2AX, CRIM1, FRK, HMGA2, and NT5DC1 were detected by WB. The apoptotic proportions of challenged SMCs were detected using FC. Detailed siRNA sequences are provided in Table S9, and antibodies used are listed in File S1.

### Phenome-wide genetic correlation analysis

We obtained the GWAS summary statistics for 2269 binary and 3 quantitative endpoints from FinnGen (data freeze 9, April 2022). Genetic correlations between AAD and these endpoints were calculated using cross-trait LDSC [73]. This method employs a weighted linear model to regress the product of *Z*-statistics for disease pairs on the LD scores of SNPs across the genome, providing unbiased estimates of genetic correlations for disease pairs, even when sample overlap exists between studies. We successfully estimated genetic correlations for 1864 pairs, and determined the significant correlations using a Bonferroni-correlated *P* value threshold of 2.68 × 10^−5^. Additionally, the *Z*-statistics of lead variants at the AAD-associated loci were extracted from the correlated diseases for further comparison.

## Ethical statement

The UKB study protocols were granted by the North West Multi-center Research Ethics Committee (Approval No. 11/NW/0382). This project was approved by the Ethics Committee of Union Hospital, Tongji Medical College, Huazhong University of Science and Technology, China (Approval No. UHCT-IEC-SOP-016-03-01).

## Supplementary Material

qzaf039_Supplementary_Data

## Data Availability

The GWAS meta-analysis summary statistics for AAD have been deposited in the Open Archive for Miscellaneous Data [74] at the National Genomics Data Center (NGDC), China National Center for Bioinformation (CNCB) (OMIX: OMIX007191), and are publicly accessible at https://ngdc.cncb.ac.cn/omix.

## CRediT author statement

**Yifan Du:** Conceptualization, Data curation, Investigation, Software, Writing – original draft. **Yunlong Guan:** Investigation, Software, Writing – original draft. **Zhonghe Shao:** Data curation, Investigation, Software, Writing – original draft. **Minghui Jiang:** Data curation, Investigation, Software. **Minghan Qu:** Investigation, Methodology. **Yifan Kong:** Investigation, Methodology. **Hongji Wu:** Investigation, Validation. **Da Luo:** Investigation, Validation. **Shu Peng:** Investigation, Validation. **Si Li:** Investigation, Visualization. **Xi Cao:** Investigation, Visualization. **Jing Chen:** Conceptualization, Resources, Writing – review & editing. **Ping Ye:** Conceptualization, Resources, Writing – review & editing. **Jiahong Xia:** Conceptualization, Funding acquisition, Project administration, Supervision, Writing – review & editing. **Xingjie Hao:** Conceptualization, Data curation, Funding acquisition, Resources, Writing – original draft, Writing – review & editing. All authors have read and approved the final manuscript.

## References

[1] Shen YH , LeMaire SA, Webb NR, Cassis LA, Daugherty A, Lu HS. Aortic aneurysms and dissections series. Arterioscler Thromb Vasc Biol 2020;40:e37–46. 32101472 10.1161/ATVBAHA.120.313991 PMC7233726

[2] Cho MJ , Lee MR, Park JG. Aortic aneurysms: current pathogenesis and therapeutic targets. Exp Mol Med 2023;55:2519–30. 38036736 10.1038/s12276-023-01130-w PMC10766996

[3] Milewicz DM , Ramirez F. Therapies for thoracic aortic aneurysms and acute aortic dissections. Arterioscler Thromb Vasc Biol 2019;39:126–36. 30651002 10.1161/ATVBAHA.118.310956 PMC6398943

[4] Huynh TT , Starr JE. Diseases of the thoracic aorta in women. J Vasc Surg 2013;57:11S–7S. 23522712 10.1016/j.jvs.2012.08.126

[5] Bossone E , Eagle KA. Epidemiology and management of aortic disease: aortic aneurysms and acute aortic syndromes. Nat Rev Cardiol 2021;18:331–48. 33353985 10.1038/s41569-020-00472-6

[6] Gao J , Cao H, Hu G, Wu Y, Xu Y, Cui H, et al The mechanism and therapy of aortic aneurysms. Signal Transduct Target Ther 2023;8:55. 36737432 10.1038/s41392-023-01325-7 PMC9898314

[7] Pinard A , Jones GT, Milewicz DM. Genetics of thoracic and abdominal aortic diseases. Circ Res 2019;124:588–606. 30763214 10.1161/CIRCRESAHA.118.312436 PMC6428422

[8] Aggarwal S , Qamar A, Sharma V, Sharma A. Abdominal aortic aneurysm: a comprehensive review. Exp Clin Cardiol 2011;16:11–5. 21523201 PMC3076160

[9] Salo JA , Soisalon-Soininen S, Bondestam S, Mattila PS. Familial occurrence of abdominal aortic aneurysm. Ann Intern Med 1999;130:637–42. 10215559 10.7326/0003-4819-130-8-199904200-00003

[10] Quintana RA , Taylor WR. Cellular mechanisms of aortic aneurysm formation. Circ Res 2019;124:607–18. 30763207 10.1161/CIRCRESAHA.118.313187 PMC6383789

[11] Roychowdhury T , Klarin D, Levin MG, Spin JM, Rhee YH, Deng A, et al Genome-wide association meta-analysis identifies risk loci for abdominal aortic aneurysm and highlights PCSK9 as a therapeutic target. Nat Genet 2023;55:1831–42. 37845353 10.1038/s41588-023-01510-y PMC10632148

[12] Klarin D , Devineni P, Sendamarai AK, Angueira AR, Graham SE, Shen YH, et al Genome-wide association study of thoracic aortic aneurysm and dissection in the Million Veteran Program. Nat Genet 2023;55:1106–15. 37308786 10.1038/s41588-023-01420-z PMC10335930

[13] Gretarsdottir S , Baas AF, Thorleifsson G, Holm H, den Heijer M, de Vries JP, et al Genome-wide association study identifies a sequence variant within the *DAB2IP* gene conferring susceptibility to abdominal aortic aneurysm. Nat Genet 2010;42:692–7. 20622881 10.1038/ng.622 PMC4157066

[14] LeMaire SA , McDonald ML, Guo DC, Russell L, Miller CC 3rd, Johnson RJ, et al Genome-wide association study identifies a susceptibility locus for thoracic aortic aneurysms and aortic dissections spanning *FBN1* at 15q21.1. Nat Genet 2011;43:996–1000. 21909107 10.1038/ng.934 PMC3244938

[15] Guo DC , Grove ML, Prakash SK, Eriksson P, Hostetler EM, LeMaire SA, et al Genetic variants in *LRP1* and *ULK4* are associated with acute aortic dissections. Am J Hum Genet 2016;99:762–9. 27569546 10.1016/j.ajhg.2016.06.034 PMC5011062

[16] Jones GT , Tromp G, Kuivaniemi H, Gretarsdottir S, Baas AF, Giusti B, et al Meta-analysis of genome-wide association studies for abdominal aortic aneurysm identifies four new disease-specific risk loci. Circ Res 2017;120:341–53. 27899403 10.1161/CIRCRESAHA.116.308765 PMC5253231

[17] Klarin D , Verma SS, Judy R, Dikilitas O, Wolford BN, Paranjpe I, et al Genetic architecture of abdominal aortic aneurysm in the Million Veteran Program. Circulation 2020;142:1633–46. 32981348 10.1161/CIRCULATIONAHA.120.047544 PMC7580856

[18] Roychowdhury T , Lu H, Hornsby WE, Crone B, Wang GT, Guo DC, et al Regulatory variants in *TCF7L2* are associated with thoracic aortic aneurysm. Am J Hum Genet 2021;108:1578–89. 34265237 10.1016/j.ajhg.2021.06.016 PMC8456156

[19] Huang H, Fang M, Jostins L, Umićević Mirkov M, Boucher G, Anderson CA, et al. Fine-mapping inflammatory bowel disease loci to single-variant resolution. Nature 2017;547:173–8. 10.1038/nature22969 PMC5511510 28658209

[20] Liu Z , Liu R, Gao H, Jung S, Gao X, Sun R, et al Genetic architecture of the inflammatory bowel diseases across East Asian and European ancestries. Nat Genet 2023;55:796–806. 37156999 10.1038/s41588-023-01384-0 PMC10290755

[21] Tachmazidou I , Hatzikotoulas K, Southam L, Esparza-Gordillo J, Haberland V, Zheng J, et al Identification of new therapeutic targets for osteoarthritis through genome-wide analyses of UK Biobank data. Nat Genet 2019;51:230–6. 30664745 10.1038/s41588-018-0327-1 PMC6400267

[22] Thibord F , Klarin D, Brody JA, Chen MH, Levin MG, Chasman DI, et al Cross-ancestry investigation of venous thromboembolism genomic predictors. Circulation 2022;146:1225–42. 36154123 10.1161/CIRCULATIONAHA.122.059675 PMC10152894

[23] Ghouse J , Tragante V, Ahlberg G, Rand SA, Jespersen JB, Leine EB, et al. Genome-wide meta-analysis identifies 93 risk loci and enables risk prediction equivalent to monogenic forms of venous thromboembolism. Nat Genet 2023;55:399–409. 10.1038/s41588-022-01286-7 36658437

[24] Boer CG , Hatzikotoulas K, Southam L, Stefánsdóttir L, Zhang Y, Coutinho de Almeida R, et al Deciphering osteoarthritis genetics across 826,690 individuals from 9 populations. Cell 2021;184:4784–818.e17. 34450027 10.1016/j.cell.2021.07.038 PMC8459317

[25] Mishra A , Malik R, Hachiya T, Jürgenson T, Namba S, Posner DC, et al Stroke genetics informs drug discovery and risk prediction across ancestries. Nature 2022;611:115–23. 36180795 10.1038/s41586-022-05165-3 PMC9524349

[26] Rashkin SR , Graff RE, Kachuri L, Thai KK, Alexeeff SE, Blatchins MA, et al Pan-cancer study detects genetic risk variants and shared genetic basis in two large cohorts. Nat Commun 2020;11:4423. 32887889 10.1038/s41467-020-18246-6 PMC7473862

[27] Sato G , Shirai Y, Namba S, Edahiro R, Sonehara K, Hata T, et al Pan-cancer and cross-population genome-wide association studies dissect shared genetic backgrounds underlying carcinogenesis. Nat Commun 2023;14:3671. 37340002 10.1038/s41467-023-39136-7 PMC10282036

[28] GTEx Consortium, et al. Genetic effects on gene expression across human tissues. Nature 2017;550:204–13. 29022597 10.1038/nature24277 PMC5776756

[29] Farh KK , Marson A, Zhu J, Kleinewietfeld M, Housley WJ, Beik S, et al Genetic and epigenetic fine mapping of causal autoimmune disease variants. Nature 2015;518:337–43. 25363779 10.1038/nature13835 PMC4336207

[30] Lu Q , Powles RL, Abdallah S, Ou D, Wang Q, Hu Y, et al Systematic tissue-specific functional annotation of the human genome highlights immune-related DNA elements for late-onset Alzheimer’s disease. PLoS Genet 2017;13:e1006933. 28742084 10.1371/journal.pgen.1006933 PMC5546707

[31] Wuttke M , Li Y, Li M, Sieber KB, Feitosa MF, Gorski M, et al A catalog of genetic loci associated with kidney function from analyses of a million individuals. Nat Genet 2019;51:957–72. 31152163 10.1038/s41588-019-0407-x PMC6698888

[32] Bycroft C , Freeman C, Petkova D, Band G, Elliott LT, Sharp K, et al The UK Biobank resource with deep phenotyping and genomic data. Nature 2018;562:203–9. 30305743 10.1038/s41586-018-0579-z PMC6786975

[33] Welter D , MacArthur J, Morales J, Burdett T, Hall P, Junkins H, et al The NHGRI GWAS Catalog, a curated resource of SNP–trait associations. Nucleic Acids Res 2014;42:D1001–6. 24316577 10.1093/nar/gkt1229 PMC3965119

[34] Reay WR , Cairns MJ. Advancing the use of genome-wide association studies for drug repurposing. Nat Rev Genet 2021;22:658–71. 34302145 10.1038/s41576-021-00387-z

[35] Pasaniuc B , Price AL. Dissecting the genetics of complex traits using summary association statistics. Nat Rev Genet 2017;18:117–27. 27840428 10.1038/nrg.2016.142 PMC5449190

[36] Kurki MI , Karjalainen J, Palta P, Sipilä TP, Kristiansson K, Donner K, et al FinnGen: unique genetic insights from combining isolated population and national health register data. medRxiv 2022;2022.03.03.22271360.

[37] Ducy P , Schinke T, Karsenty G. The osteoblast: a sophisticated fibroblast under central surveillance. Science 2000;289:1501–4. 10968779 10.1126/science.289.5484.1501

[38] Bakker MK , van der Spek RAA, van Rheenen W, Morel S, Bourcier R, Hostettler IC, et al Genome-wide association study of intracranial aneurysms identifies 17 risk loci and genetic overlap with clinical risk factors. Nat Genet 2020;52:1303–13. 33199917 10.1038/s41588-020-00725-7 PMC7116530

[39] Sakaue S , Kanai M, Tanigawa Y, Karjalainen J, Kurki M, Koshiba S, et al A cross-population atlas of genetic associations for 220 human phenotypes. Nat Genet 2021;53:1415–24. 34594039 10.1038/s41588-021-00931-x PMC12208603

[40] Saito T, , Hasegawa Y, , Ishigaki Y, , Yamada T, , Gao J, , Imai J, et al Importance of endothelial NF-κB signalling in vascular remodelling and aortic aneurysm formation. Cardiovasc Res 2013;97:106–14. 23015640 10.1093/cvr/cvs298

[41] Ren J, , Han Y, , Ren T, , Fang H, , Xu X, , Lun Y, et al AEBP1 promotes the occurrence and development of abdominal aortic aneurysm by modulating inflammation via the NF-κB pathway. J Atheroscler Thromb 2020;27:255–70. 31462616 10.5551/jat.49106 PMC7113137

[42] Gao P, , Gao P, , Zhao J, , Shan S, , Luo W, , Slivano OJ, et al MKL1 cooperates with p38MAPK to promote vascular senescence, inflammation, and abdominal aortic aneurysm. Redox Biol 2021;41:101903 33667992 10.1016/j.redox.2021.101903 PMC7937568

[43] Ortega R, , Collado A, , Selles F, , Gonzalez-Navarro H, , Sanz MJ, , Real JT, et al SGLT-2 (sodium-glucose cotransporter 2) inhibition reduces Ang II (angiotensin II)-induced dissecting abdominal aortic aneurysm in ApoE (apolipoprotein E) knockout mice. Arterioscler Thromb Vasc Biol 2019;39:1614–28. 31294626 10.1161/ATVBAHA.119.312659

[44] Sáinz-Jaspeado M , Smith RO, Plunde O, Pawelzik SC, Jin Y, Nordling S, et al Palmdelphin regulates nuclear resilience to mechanical stress in the endothelium. Circulation 2021;144:1629–45. 34636652 10.1161/CIRCULATIONAHA.121.054182 PMC8589083

[45] Wang S , Yu H, Gao J, Chen J, He P, Zhong H, et al PALMD regulates aortic valve calcification via altered glycolysis and NF-κB-mediated inflammation. J Biol Chem 2022;298:101887. 35367413 10.1016/j.jbc.2022.101887 PMC9065630

[46] Wild PS , Felix JF, Schillert A, Teumer A, Chen MH, Leening MJG, et al Large-scale genome-wide analysis identifies genetic variants associated with cardiac structure and function. J Clin Invest 2017;127:1798–812. 28394258 10.1172/JCI84840 PMC5409098

[47] Helgadottir A , Thorleifsson G, Gretarsdottir S, Stefansson OA, Tragante V, Thorolfsdottir RB, et al Genome-wide analysis yields new loci associating with aortic valve stenosis. Nat Commun 2018;9:987. 29511194 10.1038/s41467-018-03252-6 PMC5840367

[48] Thériault S , Gaudreault N, Lamontagne M, Rosa M, Boulanger MC, Messika-Zeitoun D, et al A transcriptome-wide association study identifies *PALMD* as a susceptibility gene for calcific aortic valve stenosis. Nat Commun 2018;9:988. 29511167 10.1038/s41467-018-03260-6 PMC5840407

[49] Wilkinson L , Gilbert T, Kinna G, Ruta LA, Pennisi D, Kett M, et al *Crim1*^*KST264/KST264*^ mice implicate *Crim1* in the regulation of vascular endothelial growth factor-A activity during glomerular vascular development. J Am Soc Nephrol 2007;18:1697–708. 17460146 10.1681/ASN.2006091012

[50] Wilkinson L , Gilbert T, Sipos A, Toma I, Pennisi DJ, Peti-Peterdi J, et al Loss of renal microvascular integrity in postnatal *Crim1* hypomorphic transgenic mice. Kidney Int 2009;76:1161–71. 19776720 10.1038/ki.2009.345

[51] Yim EK , Peng G, Dai H, Hu R, Li K, Lu Y, et al Rak functions as a tumor suppressor by regulating PTEN protein stability and function. Cancer Cell 2009;15:304–14. 19345329 10.1016/j.ccr.2009.02.012 PMC2673492

[52] Hua L , Zhu M, Song X, Wang J, Fang Z, Zhang C, et al FRK suppresses the proliferation of human glioma cells by inhibiting cyclin D1 nuclear accumulation. J Neurooncol 2014;119:49–58. 24792491 10.1007/s11060-014-1461-y

[53] Ligthart S , Vaez A, Võsa U, Stathopoulou MG, de Vries PS, Prins BP, et al Genome analyses of > 200,000 individuals identify 58 loci for chronic inflammation and highlight pathways that link inflammation and complex disorders. Am J Hum Genet 2018;103:691–706. 30388399 10.1016/j.ajhg.2018.09.009 PMC6218410

[54] Richardson TG , Sanderson E, Palmer TM, Ala-Korpela M, Ference BA, Davey Smith G, et al Evaluating the relationship between circulating lipoprotein lipids and apolipoproteins with risk of coronary heart disease: a multivariable Mendelian randomisation analysis. PLoS Med 2020;17:e1003062. 32203549 10.1371/journal.pmed.1003062 PMC7089422

[55] Summer H , Li O, Bao Q, Zhan L, Peter S, Sathiyanathan P, et al HMGA2 exhibits dRP/AP site cleavage activity and protects cancer cells from DNA-damage-induced cytotoxicity during chemotherapy. Nucleic Acids Res 2009;37:4371–84. 19465398 10.1093/nar/gkp375 PMC2715238

[56] Nishino J , Kim I, Chada K, Morrison SJ. Hmga2 promotes neural stem cell self-renewal in young but not old mice by reducing p16^Ink4a^ and p19^Arf^ expression. Cell 2008;135:227–39. 18957199 10.1016/j.cell.2008.09.01 PMC2582221

[57] Vasan RS , Glazer NL, Felix JF, Lieb W, Wild PS, Felix SB, et al Genetic variants associated with cardiac structure and function: a meta-analysis and replication of genome-wide association data. JAMA 2009;302:168–78. 19584346 10.1001/jama.2009.978-a PMC2975567

[58] Hoffmann TJ , Ehret GB, Nandakumar P, Ranatunga D, Schaefer C, Kwok PY, et al Genome-wide association analyses using electronic health records identify new loci influencing blood pressure variation. Nat Genet 2017;49:54–64. 27841878 10.1038/ng.3715 PMC5370207

[59] Shungin D , Winkler TW, Croteau-Chonka DC, Ferreira T, Locke AE, Mägi R, et al New genetic loci link adipose and insulin biology to body fat distribution. Nature 2015;518:187–96. 25673412 10.1038/nature14132 PMC4338562

[60] Guo Y , Gong Y, Shi G, Yang K, Pan C, Li M, et al Single-nucleotide polymorphisms in the *TSPYL-4* and *NT5DC1* genes are associated with susceptibility to chronic obstructive pulmonary disease. Mol Med Rep 2012;6:631–8. 22736055 10.3892/mmr.2012.964

[61] Yu X , Sun R, Yang X, He X, Guo H, Ou C. The NT5DC family: expression profile and prognostic value in pancreatic adenocarcinoma. J Cancer 2023;14:2274–88. 37576396 10.7150/jca.85811 PMC10414034

[62] Hao X , Shao Z, Zhang N, Jiang M, Cao X, Li S, et al Integrative genome-wide analyses identify novel loci associated with kidney stones and provide insights into its genetic architecture. Nat Commun 2023;14:7498. 37980427 10.1038/s41467-023-43400-1 PMC10657403

[63] Chang CC , Chow CC, Tellier LC, Vattikuti S, Purcell SM, Lee JJ. Second-generation PLINK: rising to the challenge of larger and richer datasets. Gigascience 2015;4:7. 25722852 10.1186/s13742-015-0047-8 PMC4342193

[64] Mbatchou J , Barnard L, Backman J, Marcketta A, Kosmicki JA, Ziyatdinov A, et al Computationally efficient whole-genome regression for quantitative and binary traits. Nat Genet 2021;53:1097–103. 34017140 10.1038/s41588-021-00870-7

[65] Willer CJ , Li Y, Abecasis GR. METAL: fast and efficient meta-analysis of genomewide association scans. Bioinformatics 2010;26:2190–1. 20616382 10.1093/bioinformatics/btq340 PMC2922887

[66] Yang J , Lee SH, Goddard ME, Visscher PM. GCTA: a tool for genome-wide complex trait analysis. Am J Hum Genet 2011;88:76–82. 21167468 10.1016/j.ajhg.2010.11.011 PMC3014363

[67] Adlam D , Berrandou TE, Georges A, Nelson CP, Giannoulatou E, Henry J, et al Genome-wide association meta-analysis of spontaneous coronary artery dissection identifies risk variants and genes related to artery integrity and tissue-mediated coagulation. Nat Genet 2023;55:964–72. 37248441 10.1038/s41588-023-01410-1 PMC10260398

[68] Finucane HK , Reshef YA, Anttila V, Slowikowski K, Gusev A, Byrnes A, et al Heritability enrichment of specifically expressed genes identifies disease-relevant tissues and cell types. Nat Genet 2018;50:621–9. 29632380 10.1038/s41588-018-0081-4 PMC5896795

[69] Finucane HK , Bulik-Sullivan B, Gusev A, Trynka G, Reshef Y, Loh PR, et al Partitioning heritability by functional annotation using genome-wide association summary statistics. Nat Genet 2015;47:1228–35. 26414678 10.1038/ng.3404 PMC4626285

[70] Watanabe K , Taskesen E, van Bochoven A, Posthuma D. Functional mapping and annotation of genetic associations with FUMA. Nat Commun 2017;8:1826. 29184056 10.1038/s41467-017-01261-5 PMC5705698

[71] Gusev A , Ko A, Shi H, Bhatia G, Chung W, Penninx BWJH, et al Integrative approaches for large-scale transcriptome-wide association studies. Nat Genet 2016;48:245–52. 26854917 10.1038/ng.3506 PMC4767558

[72] Butler A , Hoffman P, Smibert P, Papalexi E, Satija R. Integrating single-cell transcriptomic data across different conditions, technologies, and species. Nat Biotechnol 2018;36:411–20. 29608179 10.1038/nbt.4096 PMC6700744

[73] Bulik-Sullivan B , Finucane HK, Anttila V, Gusev A, Day FR, Loh PR, et al An atlas of genetic correlations across human diseases and traits. Nat Genet 2015;47:1236–41 26414676 10.1038/ng.3406 PMC4797329

[74] Chen T , Chen X, Zhang S, Zhu J, Tang B, Wang A, et al The Genome Sequence Archive Family: toward explosive data growth and diverse data types. Genomics Proteomics Bioinformatics 2021;19:578–83. 34400360 10.1016/j.gpb.2021.08.001 PMC9039563

