# Supplementary material for: Integrative Genome-wide Association Meta-analysis of Aortic Aneurysm and Dissection Identifies Five Novel Genes
Source: Genomics Proteomics Bioinformatics. 2025 Apr 29;23(5):qzaf039. doi: 10.1093/gpbjnl/qzaf039 (PMC12902790; doi:10.1093/gpbjnl/qzaf039)
Supplement: qzaf039_Supplementary_Data [file qzaf039_supplementary_data.zip › File S1.docx]

**File S1 The details of supplementary methods**

**Replication in the BioBank of Japan and Michigan Genomics Initiative**

To validate the novel loci identified in our genome-wide association study (GWAS) meta-analysis, we queried these signals in aortic aneurysm and dissectionAAD GWAS datasets from the Biobank of Japan (BBJ) and the Michigan Genomics Initiative (MGI). BBJ is a biobank that collected DNA and serum samples from 12 medical institutions in Japan, recruiting around 200,000 participants, primarily of Japanese ancestry. 220 deep-phenotype GWASs in BBJ were conducted and the summary statistics were deposited at BBJ PheWeb (https://pheweb.jp/). The AAD GWAS in BBJ contained 1155 cases and 172,446 controls. MGI is a biobank comprising Michigan Medicine patients. The MGI PheWeb (https://pheweb.org/MGI/) provides GWAS results for 1542 phenotypes derived from electronic health records, including approximately 51.8 million imputed variants in 51,583 individuals of European-ancestry. The AAD GWAS in MGI included 1846 cases and 41,836 controls.

**Candidate gene mapping and prioritization**

*Position mapping*

For each locus, the nearest protein-coding gene to the lead variant was mapped as the candidate gene.

*Nonsynonymous variant mapping*

All significant (*P* < 5 × 10^−8^) variants were annotated using ANNOVAR [1]. Then we selected all nonsynonymous variants to prioritize the genes.

*Artery-specific expression quantitative trait loci mapping*

We conducted gene mapping based on the expression quantitative trait loci (eQTL) of aorta artery, coronary artery, and tibial artery from the genotype-tissue expression (GTEx) v.8 project [2]. When the gene was mapped by multiple eQTL variants at the susceptibility locus, the GWAS lead variant at this locus or the variant with the smallest eQTL *P* values was reported for each tissue.

*Artery-specific transcriptome-wide association study and colocalisation*

Transcriptome-wide association study (TWAS) was conducted using the functional summary-based imputation (FUSION) [3] pipelines. We focused on artery-specific eQTL of European ancestry from the GTEx v.8 project, including aorta artery (n = 329), coronary artery (n = 175), and tibial artery (n = 476) [2]. To boost the TWAS power, the aggregated Cauchy association test (ACAT) [4] was applied to combine *P* values for each gene across three different arteries. A total of 15,051 genes were examined. A gene expression was assumed to be associated with AAD if the ACAT *P* value was less than 3.32 × 10^−6^ (0.05/15,051). We also performed Bayesian colocalisation analysis [5] to investigate whether a causal variant influence both AAD and gene expression, which was also implemented in the FUSION [3] pipelines. AAD and artery-specific gene expression were assumed to be influenced by a shared causal variant if the posterior probability of colocalization was larger than 0.8.

**Functional annotation and drug targets for the prioritized genes**

We investigated the function and annotation of the prioritized genes using GENE2FUNC in the functional mapping and annotation of genetic associations (FUMA) platform [6]. The gene set enrichment analyses were conducted, including differently expressed gene in multiple tissues from the GTEx v.8 project [2], Reactome pathway, Kyoto Encyclopedia of Genes and Genomes (KEGG) pathway, Gene Ontology (GO) biological processes (BP) pathway, GO cellular components (CC) pathway, and GO molecular functions (MF) pathway. The known targets of drugs for prioritized genes were linked to DrugBank [7] (https://go.drugbank.com/).

Gene set enrichment analysis showed that these prioritized genes were enriched in the up-regulated expressed gene set of aorta artery, coronary artery, and tibial artery (Figure S5). These genes were also enriched in 12 additional pathways, including “response to growth factor” from GO BP, “elastic fiber formation” from Reactome, and “TGF-β signaling” from KEGG (Table S5). In the DrugBank database, 11 prioritized genes were targeted by 36 drugs (Table S6). For example, the novel gene *FRK* is targeted by dasatinib (DB01254), regorafenib (DB08896), and fostamatinib (DB12010). Fostamatinib also targets two other prioritized genes *MAP2K5* and *CLK3.* Fostamatinib is a spleen tyrosine kinase inhibitor used to treat chronic immune thrombocytopenia by blocking the activity of the enzyme spleen tyrosine kinase and reducing the immune system’s destruction of platelets. In addition, the recent study suggested that ceramide degradation mediated by alkaline ceramidase in platelets demonstrated anti-inflammatory properties and reduced abdominal AAD (AAAD) formation [8]. The known gene *PDE3A* was targeted by 11 drugs. *PDE3A* encodes a member of the cGMP-inhibited cyclic nucleotide phosphodiesterase family, which regulates platelet aggregation and plays crucial roles in cardiovascular function by modulating the contraction and relaxation of vascular smooth muscles [9]. Among the 11 drugs targeting *PDE3A*, seven are involved in vasodilation and vasoconstriction. These prioritized genes provided the target medications that could alleviate vascular wall stress, mitigate damage to the vascular wall, and potentially reduce the incidence of AAD.

To further explore the potential of prioritized genes as drug targets for AAD, we performed a drug target Mendelian randomization (MR) analysis on one of these genes, namely *LPA*. UK Biobank Pharma Proteomics Project (UKB-PPP) is a large proteomic study that measured 2941 protein analytes (PMID: 37794186). We identified the protein quantitative trait loci (pQTLs) for LPA in UKB-PPP with the following criteria: (1) *cis*-region (± 500 kb) of gene *LPA*; (2) minor allele frequency > 0.05; (3) significantly associated with the level of plasma LPA (*P* < 5 × 10^−8^); and (4) passed a stringent linkage disequilibrium (LD) with *r^2^* threshold of 0.001. Finally, four single nucleotide polymorphisms (SNPs) (*i.e.*, rs4646272, rs62440924, rs55730499, and rs9355839) were included as pQTLs for LPA. Employing our GWAS summary data as the outcome, the inverse-variance weighted (IVW)-MR analysis revealed that the risk of AAD increased by 14% for each standard deviation increase in plasma LPA level [odds ratio (OR): 1.14; 95% confidence interval (CI): 1.09–1.20]. This implies that the targeted drugs for *LPA* gene, such as DB00513, could potentially lower the risk of AAD.

**Causal cardiovascular risk factors for AAD**

MR analyses based on GWAS summary statistics were performed to detect the potential causal relationships between lipids (*i.e.*, low-density lipoprotein (LDL), high-density lipoprotein (HDL), total cholesterol (TC), and triglycerides (TG)), blood pressure (*i.e.*, systolic blood pressure (SBP), diastolic blood pressure (DBP), and pulse pressure (PP)), body mass index (BMI), and AAD using the R packages TwoSampleMR [10]. MR-Egger method [11] which is robust to the horizontal pleiotropic effect was chosen as the main analysis. Other methods, including IVW [12] and weighted median [13] were applied as sensitivity analyses. For instrumental variable (IV) selection, independent and significant variants (*P* < 5 × 10^-8^ and LD *r*^2^ < 0.001 within 10 Mb based on the 1000 Genomes Project phase 3 European ancestry reference panel) associated with the exposure were kept. We additionally applied the MR pleiotropy residual sum and outlier (MR-PRESSO) method [14] to detect and remove horizontal pleiotropic SNPs (*P* < 0.05). The genetic correlations between these exposures and AAD were also estimated by cross-trait linkage disequilibrium score (LDSC) [15]. The GWAS summary statistics of exposures were from the Million Veteran Program (database of genotypes and phenotypes (dbGaP) accession number is phs001672).

Among these cardiovascular risk factors, genetic correlations with AAD were observed (*P* < 0.05), except for SBP and PP (Figure S7). MR analyses were performed using the MR-Egger method, which is robust against pleiotropic effect. The results indicated that the genetically elevated levels of LDL, TC, TG, and BMI were associated with increased odds of AAD. Conversely, genetically elevated levels of HDL and PP were found to have protective effects against AAD after Bonferroni correction (*P* < 0.006) (Figure S7). These significant associations were remained robust in the sensitivity analyses performed using IVW and weighted median methods (Figure S8).

Interestingly, prior MR studies have indicated that lipids are causally associated with AAAD subtype of AAD [16], whereas they do not appear to be associated with thoracic AAD (TAAD) [17]. In observational studies, hypercholesterolemia and atherosclerosis have been recognized as risk factors for both TAAD and AAAD [18,19]. Therefore, the causal relationship between lipids and TAAD requires further investigation using larger sample sizes for both outcome and exposure. In addition, DBP and PP were the causal blood pressure traits for TAAD [17], with DBP also being a causal factor for AAAD according to an MR study of SBP and DBP on AAAD [20]. However, PP alone was identified as the causal factor for AAD. It is worth noting that PP is influenced by the structural and functional properties of the aorta [21]. MR studies have suggested that elevated genetically predicted PP is associated with reduced ascending aorta diameter [22,23]. In health population, higher PP usually reflects lower peripheral circulating pressure, and thus the aortic root does not need to be stressed too much to pump blood out during diastole, which can further relieve the burden of the aorta and reduce the incidence of AAD. The relationships between BMI and AAD, as well as its subtypes have not been extensively studied. The role of high BMI as a risk factor for AAD might be mediated through the lipid levels.

**Antibodies used for the immunoblot analyses and immunostaining**

| **Antibody** | **Cat No.** | **Manufacturer** | **MW (kDa)** | **Application** |
| --- | --- | --- | --- | --- |
| P38 | 9212 | CST (Danvers, MA) | 40 | IHC, WB |
| p-P38 | 4511 | CST | 43 | WB, IHC, IP, IF |
| P65 | 8242 | CST | 65 | WB, IHC, IP, IF |
| p-P65 | 3033 | CST | 65 | WB, IP, IF |
| Cleaved Caspase-3 | 25128-1-AP | Proteintech (China) | 17, 19 | WB, IHC, IF, ELISA |
| γ-H2AX | AF3187 | Affinity (China) | 15 | WB, IHC |
| Calponin | 13938-1-AP | Proteintech | 35 | WB, IP, IHC, IF, FC, ELISA |
| SM22-α | 10493-1-AP | Proteintech | 22 | WB, IHC, IF, FC, ELISA |
| β-actin | 81115-1-RR | Proteintech | 42 | WB, IHC, IF, ELISA |
| ICAM-1 | 10831-1-AP | Proteintech | 90 | WB, IP, IHC, IF, FC |
| VCAM-1 | 11444-1-AP | Proteintech | 110 | WB, IHC, IF, FC, ELISA |
| PALMD | 16531-1-AP | Proteintech | 80 | WB, IHC, IF, IP, ELISA |
| CRIM1 | K009538P | Solarbio (China) | 110 | IF, IHC, ELISA |
| FRK | 16197-1-AP | Proteintech | 54–57 | WB, IHC, IF, FC, ELISA |
| HMGA2 | 20795-1-AP | Proteintech | 18–20 | WB, IHC, IF, IP, ELISA |
| NT5DC1 | 24102-1-AP | Proteintech | 52 | IF, WB, ELISA |
| NT5DC1 | K113036P | Solarbio | 52 | WB |

*Note*: The information of antibodies used in *ex vivo* and *in vitro* experiment. MW, molecular weight; WB, Western blot; IHC, immunohistochemistry; IF, immunofluorescence; FC, flow cytometry; ELISA, enzyme-linked immunosorbent assay; IP, Immunoprecipitation.

**Reference**

[1] Wang K, Li M, Hakonarson H. ANNOVAR: functional annotation of genetic variants from high-throughput sequencing data. Nucleic Acids Res 2010;38:e164.

[2] GTEx Consortium. The Genotype-Tissue Expression (GTEx) pilot analysis: multitissue gene regulation in humans. Science 2015;348:648–60.

[3] Gusev A, Ko A, Shi H, Bhatia G, Chung W, Penninx BW, et al. Integrative approaches for large-scale transcriptome-wide association studies. Nat Genet 2016;48:245–52.

[4] Liu Y, Chen S, Li Z, Morrison AC, Boerwinkle E, Lin X. ACAT: a fast and powerful *P* value combination method for rare-variant analysis in sequencing studies. Am J Hum Genet 2019;104:410–21.

[5] Giambartolomei C, Vukcevic D, Schadt EE, Franke L, Hingorani AD, Wallace C, et al. Bayesian test for colocalisation between pairs of genetic association studies using summary statistics. PLoS Genet 2014;10:e1004383.

[6] Watanabe K, Taskesen E, Bochoven AV, Posthuma D. Functional mapping and annotation of genetic associations with FUMA. Nat Commun 2017;8:1826.

[7] Wishart DS, Feunang YD, Guo AC, Lo EJ, Marcu A, Grant JR, et al. DrugBank 5.0: a major update to the DrugBank database for 2018. Nucleic Acids Res 2017;46:D1074–82.

[8] Zhang X, Gong Z, Shen Y, Cai Z, Yang L, Zhang T, et al. Alkaline ceramidase 1-mediated platelet ceramide catabolism mitigates vascular inflammation and abdominal aortic aneurysm formation. Nat Cardiovasc Res 2023;2:1173–89.

[9] Chen J, Liu N, Huang Y, Wang Y, Sun Y, Wu Q, et al. Structure of PDE3A–SLFN12 complex and structure-based design for a potent apoptosis inducer of tumor cells. Nat Commun 2021;12:6204.

[10] Hemani G, Zheng J, Elsworth B, Wade KH, Haberland V, Baird D, et al. The MR-Base platform supports systematic causal inference across the human phenome. Elife 2018;7:e34408.

[11] Bowden J, Davey Smith G, Burgess S. Mendelian randomization with invalid instruments: effect estimation and bias detection through Egger regression. Int J Epidemiol 2015;44:512–25.

[12] Burgess S, Butterworth A, Thompson SG. Mendelian randomization analysis with multiple genetic variants using summarized data. Genet Epidemiol 2013;37:658–65.

[13] Bowden J, Davey Smith G, Haycock PC, Burgess S. Consistent estimation in Mendelian randomization with some invalid instruments using a weighted median estimator. Genet Epidemiol 2016;40:304–14.

[14] Verbanck M, Chen CY, Neale B, Do R. Detection of widespread horizontal pleiotropy in causal relationships inferred from Mendelian randomization between complex traits and diseases. Nat Genet 2018;50:693–8.

[15] Bulik-Sullivan B, Finucane HK, Anttila V, Gusev A, Day FR, Loh PR, et al. An atlas of genetic correlations across human diseases and traits. Nat Genet 2015;47:1236–41.

[16]Roychowdhury T, Klarin D, Levin MG, Spin JM, Rhee YH, Deng A, et al. Genome-wide association meta-analysis identifies risk loci for abdominal aortic aneurysm and highlights PCSK9 as a therapeutic target. Nat Genet 2023;55:1831–42.

[17] Klarin D, Devineni P, Sendamarai AK, Angueira AR, Graham SE, Shen YH, et al. Genome-wide association study of thoracic aortic aneurysm and dissection in the Million Veteran Program. Nat Genet 2023;55:1106–15.

[18] Isselbacher EM, Preventza O, Hamilton Black J 3rd, Augoustides JG, Beck AW, Bolen MA, et al. 2022 ACC/AHA Guideline for the Diagnosis and Management of Aortic Disease: a report of the American Heart Association/American College of Cardiology Joint Committee on Clinical Practice Guidelines. Circulation 2022;146:e334–482.

[19] Isselbacher EM. Thoracic and abdominal aortic aneurysms. Circulation 2005;111:816–28.

[20] Klarin D, Verma SS, Judy R, Dikilitas O, Wolford BN, Paranjpe I, et al. Genetic architecture of abdominal aortic aneurysm in the Million Veteran Program. Circulation 2020;142:1633–46.

[21] Farasat SM, Morrell CH, Scuteri A, Ting CT, Yin FC, Spurgeon HA, et al. Pulse pressure is inversely related to aortic root diameter implications for the pathogenesis of systolic hypertension. Hypertension 2008;51:196–202.

[22] Tcheandjieu C, Xiao K, Tejeda H, Lynch JA, Ruotsalainen S, Bellomo T, et al. High heritability of ascending aortic diameter and trans-ancestry prediction of thoracic aortic disease. Nat Genet 2022;54:772–82.

[23] DePaolo J, Levin MG, Tcheandjieu C, Priest JR, Gill D, Burgess S, et al. Relationship between ascending thoracic aortic diameter and blood pressure: a Mendelian randomization study. Arterioscler Thromb Vasc Biol 2023;43:359–66.
