# Supplementary material for: Integrative Genome-wide Association Meta-analysis of Aortic Aneurysm and Dissection Identifies Five Novel Genes
Source: Genomics Proteomics Bioinformatics. 2025 Apr 29;23(5):qzaf039. doi: 10.1093/gpbjnl/qzaf039 (PMC12902790; doi:10.1093/gpbjnl/qzaf039)
Supplement: qzaf039_Supplementary_Data [file qzaf039_supplementary_data.zip › supplementary material captions.docx]

**Supplementary material**

**File S1 The details of supplementary methods**

**Figure S1 Analysis workflow of the integrative GWAS on AAD**

Overview of our pipeline for the GWAS meta-analysis and integrative analysis of GWAS hits with multi-omics and multiple traits. See Materials and methods and Results for more details. IVW, inverse-variance weighted; eQTL, expression quantitative trait loci.

**Figure S2 The effect comparison between our meta-analysis, BBJ, and MGI result**

BBJ includes 1155 AAD cases and 172,446 controls. MGI includes 1846 AAD cases and 41,836 controls. The meta-analysis of BBJ and MGI was conducted using inverse variance-based method. The horizontal and vertical lines indicate that OR is 1, and the slope of the diagonal line is 1. BBJ, BioBank of Japan; MGI, Michigan Genomics Initiative.

**Figure S3 The expression of prioritized genes at the novel loci in different tissues**

**A.** *PALMD*. **B.** *CRIM1*. **C**. *FRK*. **D.** *HMGA2*. **E.** *NT5DC1*. The Y-axis represents the expression level with total counts per million (TPM). The X-axis represents 54 tissues from the GTEx v.8 project. The color represents different tissues. These figures were downloaded from the GTEx portal https://www.gtexportal.org/home/. TPM, total counts per million; GTEx, genotype-tissue expression project.

**Figure S4 Relevant tissue and cell types for abdominal aortic aneurysm**

**A.** The cell type landscape in human abdominal aortic aneurysm based on scRNA-seq. **B.** Dot plots for five prioritized genes at the novel loci based on the gene expression in different cell type. **C.** Detailed subcluster analysis of VSMC/EC/fibroblast cluster in A. **D.** and **E.** Dot plots and feature plots for five prioritized genes at the novel loci based on the gene expression in different cell type in C. EC, endothelial cell.

**Figure S5 The tissue enrichment of the prioritized genes based on the gene expression in different tissues**

The X-axis represents 54 tissues from the GTEx v.8 project. Red color indicates significant tissue enrichment with a *P* value passed the Bonferroni-corrected significance level at 0.0009. DEG, differently expressed gene.

**Figure S6 Quantification of each panel in the Figure 4**

All experiments were repeated at least for 3 times. All data represent independent data points but not technical replicates. Data are presented as the mean ± SD. For comparisons of 2 groups, Student unpaired two-tailed *t*-test was used for normally distributed data. *, *P* < 0.05; **, *P* < 0.01; ***, *P* < 0.001; ****, *P* < 0.000; ns, not significant. SD, standard deviation.

**Figure S7 MR analyses of cardiovascular risk factors**

The left panel shows the genetic correlation between cardiovascular risk factors and AAD, and the right panel shows the causal effects of cardiovascular risk factors on AAD based on MR-Egger method. *r*_g_ is the genetic correlation. SBP, systolic blood pressure; DBP, diastolic blood pressure; PP, pulse pressure; HDL, high-density lipoprotein; LDL, low-density lipoprotein; TC, total cholesterol; TG, triglyceride; BMI, body mass index; MR, Mendelian randomization.

**Figure S8 The forest plot of MR analyses using three different methods**

**Table S1 AAD associated significant nonsynonymous variants**

**Table S2 AAD putative genes prioritized by artery-specific eQTL**

**Table S3 AAD putative genes prioritized by artery-specific TWAS**

**Table S4 Summary of the AAD prioritized genes by four approaches**

**Table S5 Gene-set enrichment analysis for the AAD prioritized genes based on hypergeometric test in FUMA**

**Table S6 Targeted drugs for the AAD prioritized genes in the DrugBank database**

**Table S7 Phenome-wide genetic correlation with AAD in FinnGen study**

**Table S8 Association of AAD lead variants with the genetic correlated diseases**

**Table S9 The information of siRNA**
