# Supplementary figures and images for: Integrative Genome-wide Association Meta-analysis of Aortic Aneurysm and Dissection Identifies Five Novel Genes
Source: Genomics Proteomics Bioinformatics. 2025 Apr 29;23(5):qzaf039. doi: 10.1093/gpbjnl/qzaf039 (PMC12902790; doi:10.1093/gpbjnl/qzaf039)

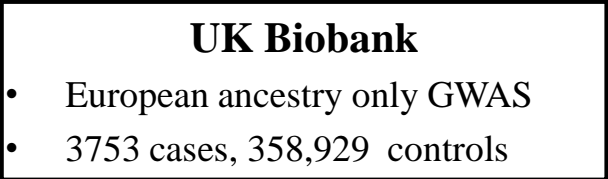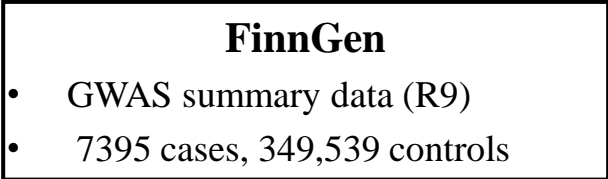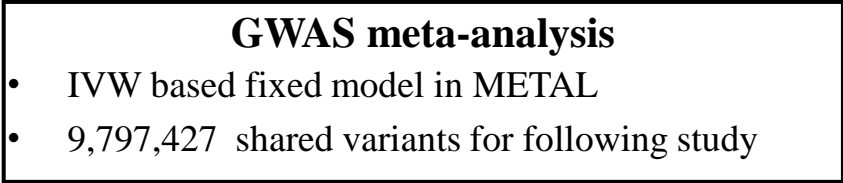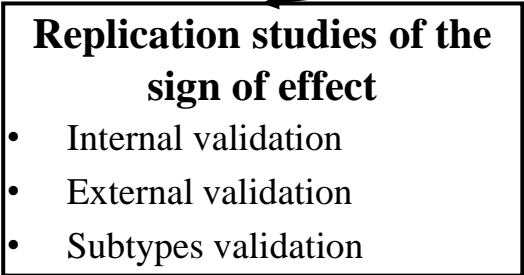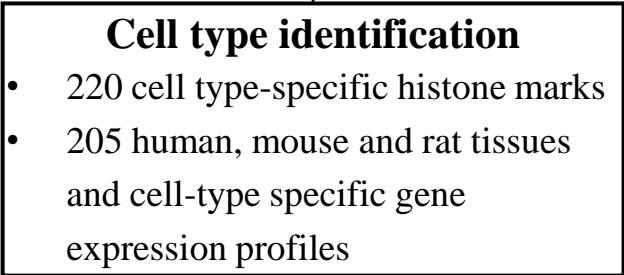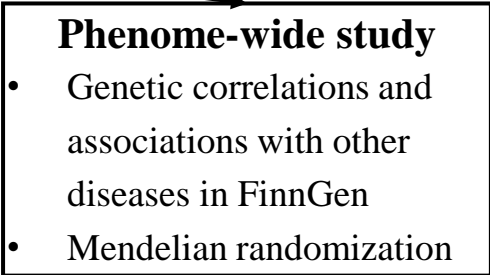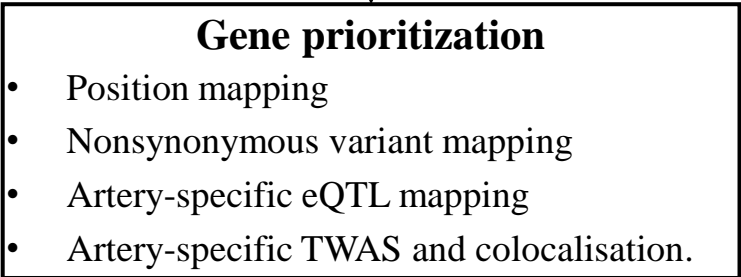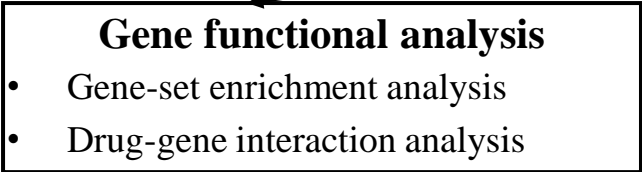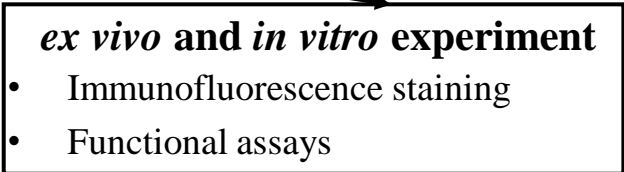

Supplement: qzaf039_Supplementary_Data [file qzaf039_supplementary_data.zip › Figure S1.pdf]

**A**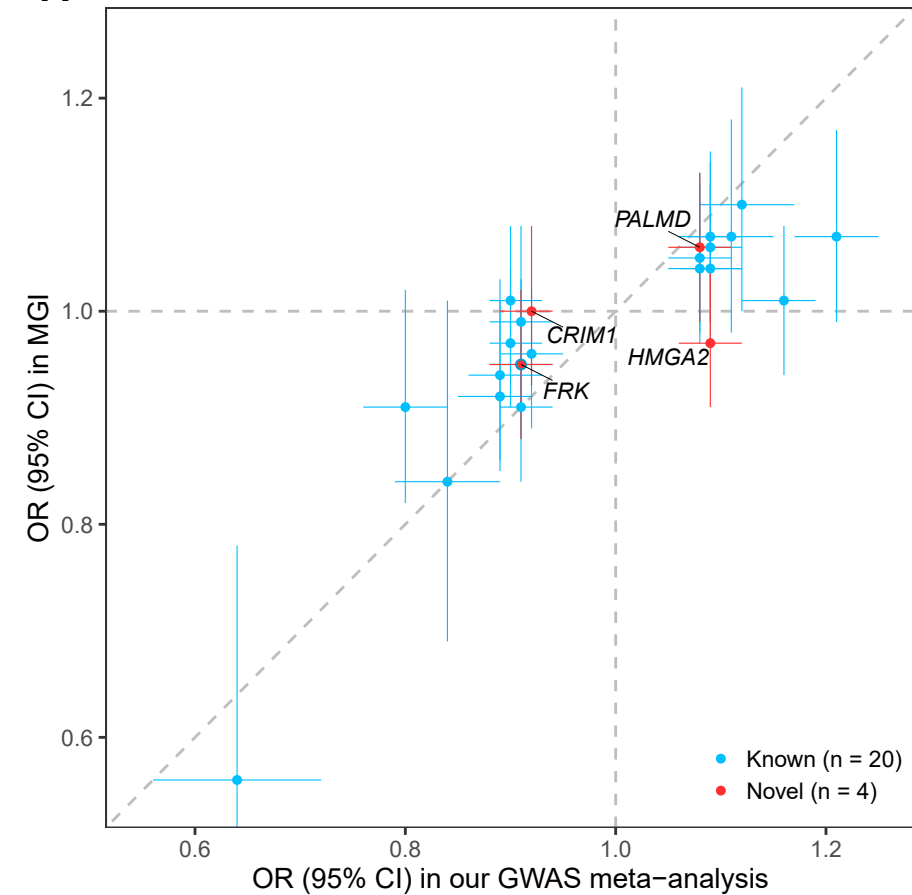**B**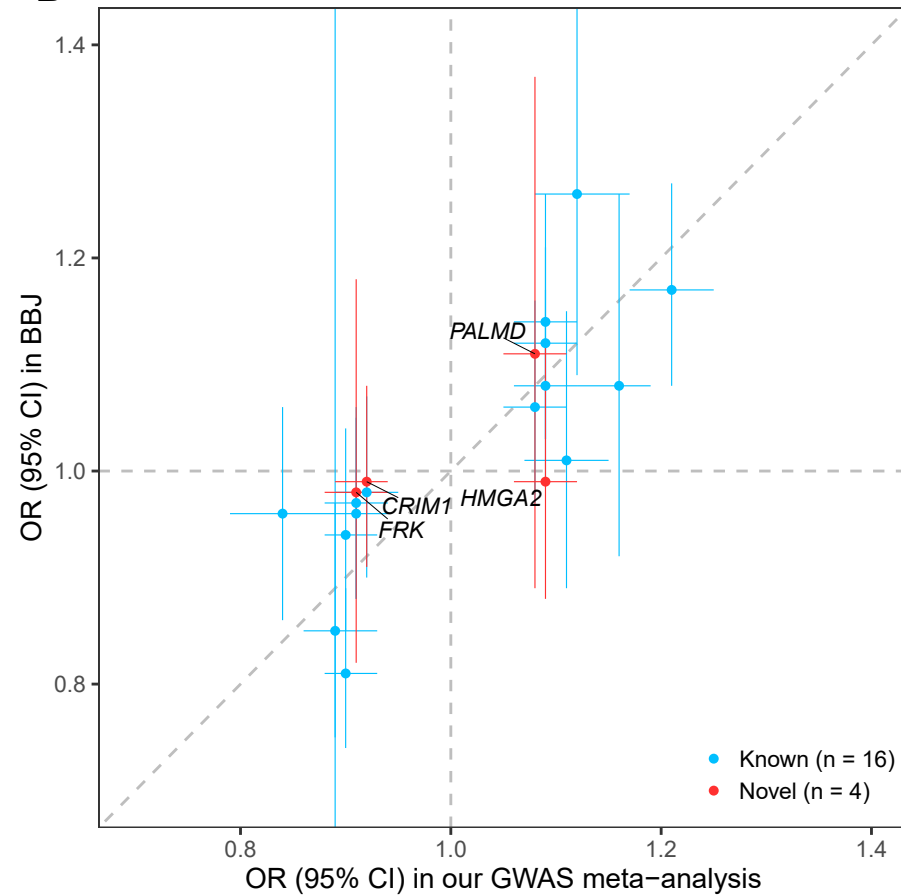**C**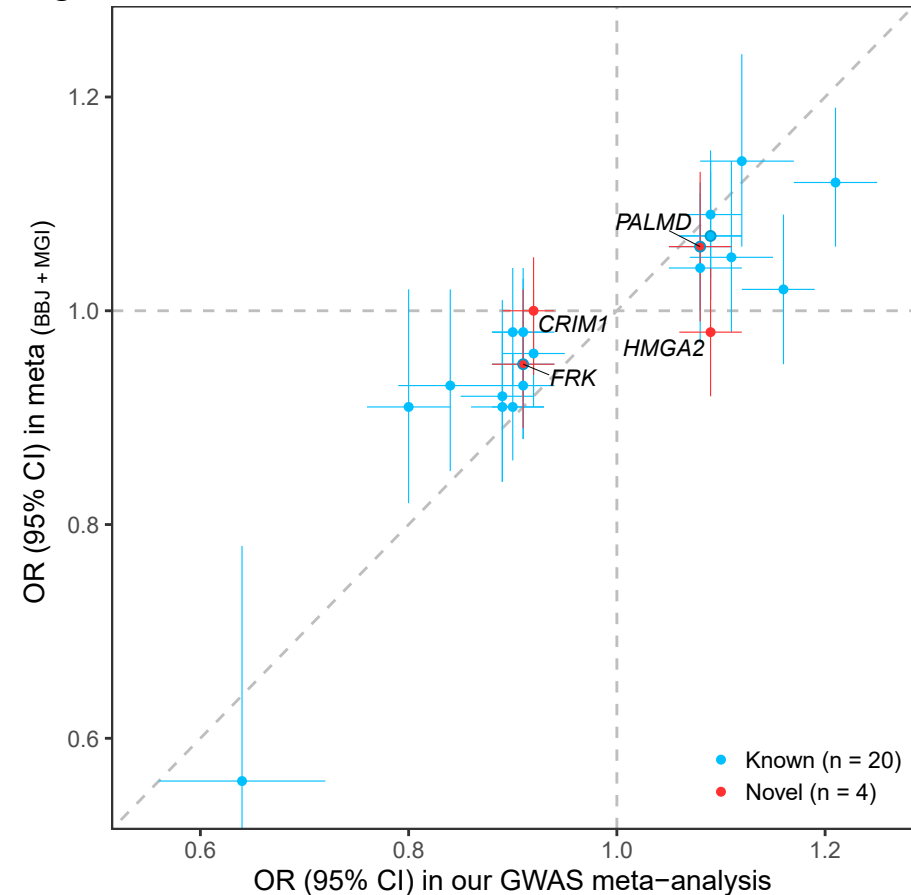

Supplement: qzaf039_Supplementary_Data [file qzaf039_supplementary_data.zip › Figure S2.pdf]

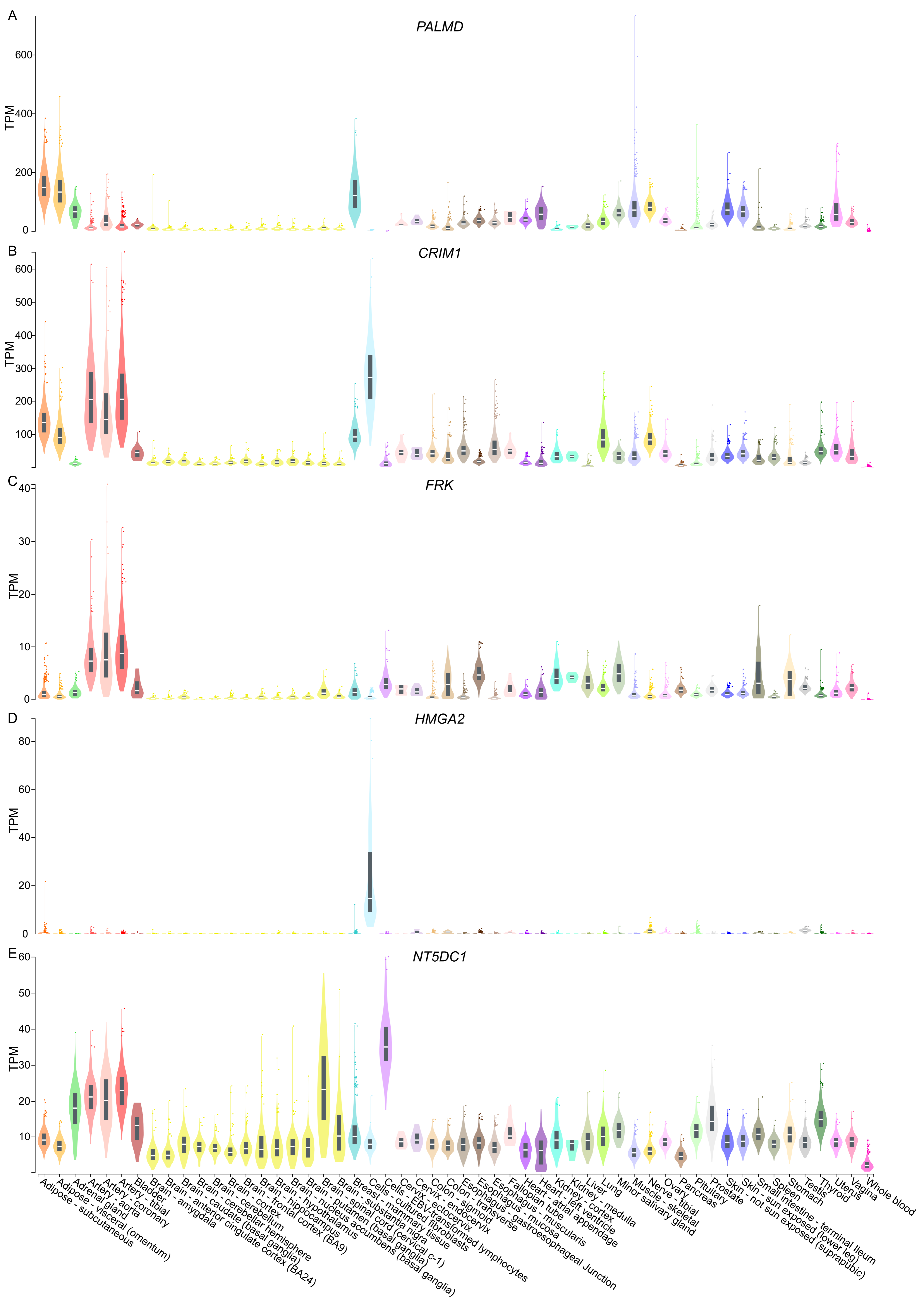

Supplement: qzaf039_Supplementary_Data [file qzaf039_supplementary_data.zip › Figure S3.pdf]

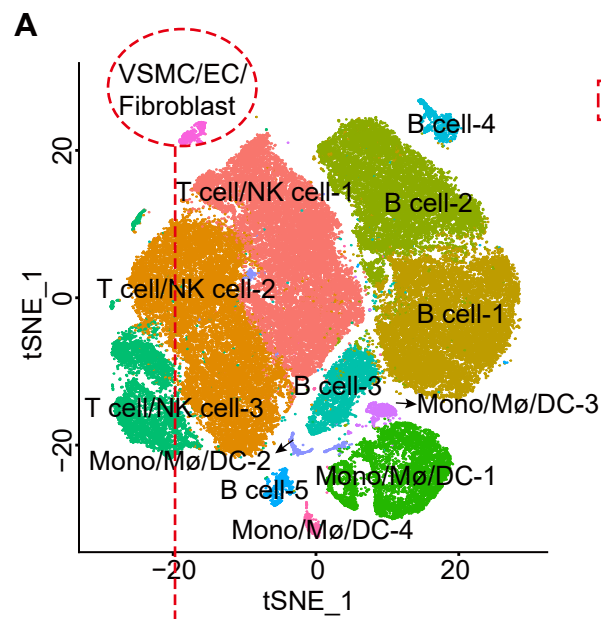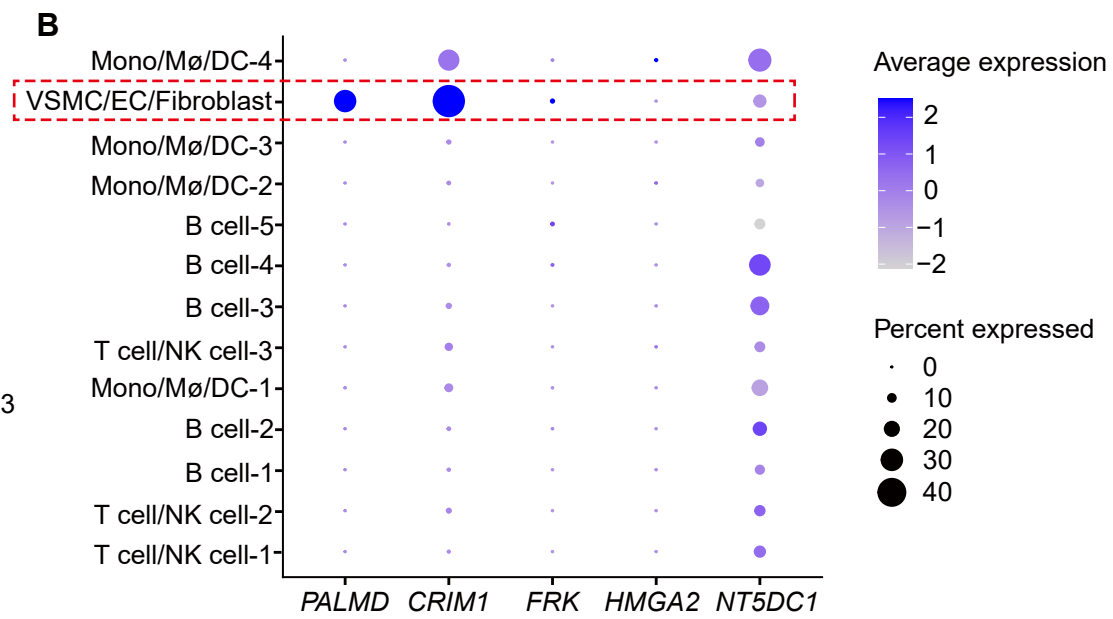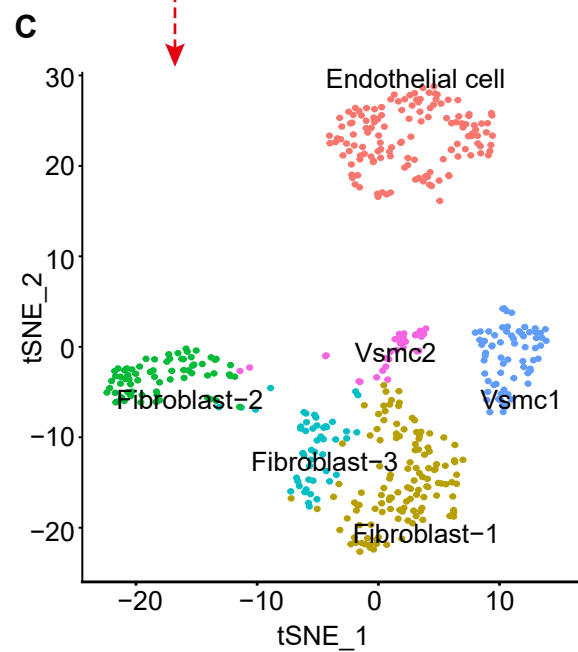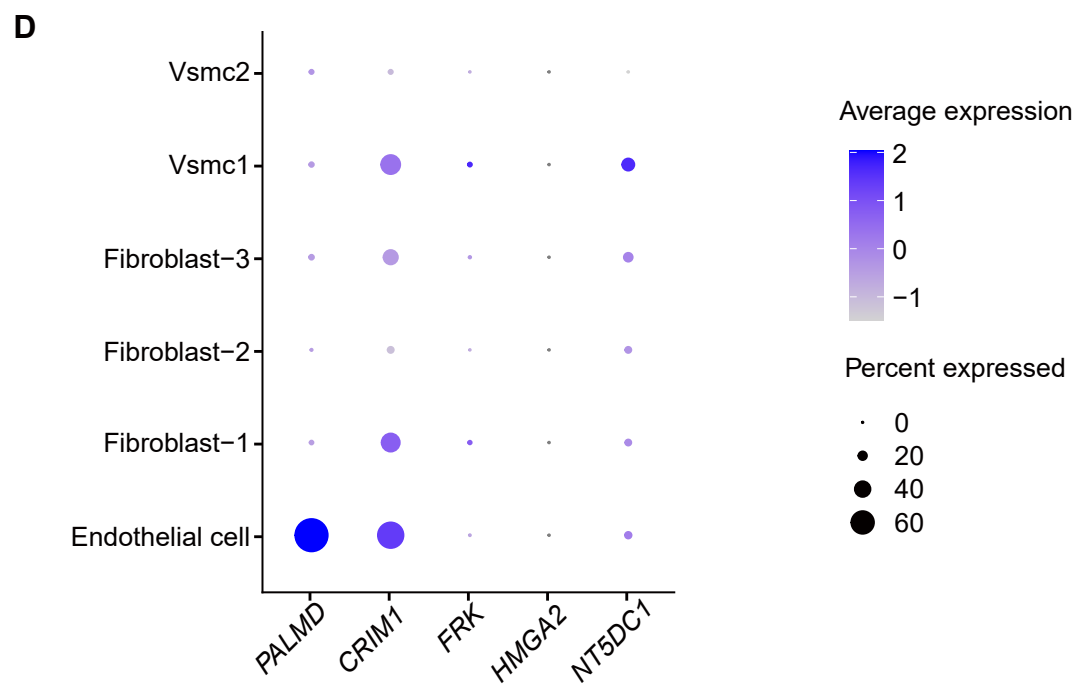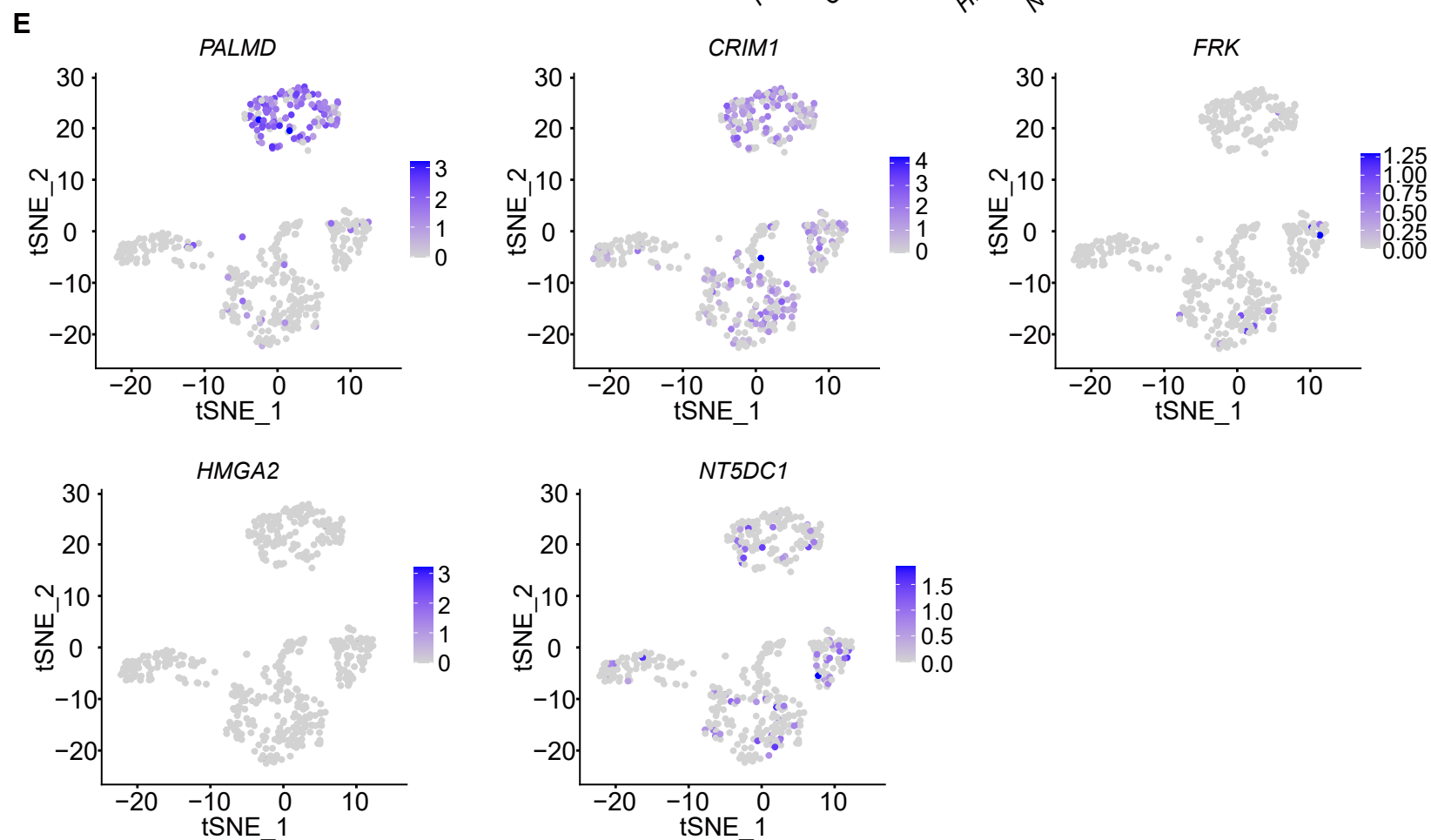

Supplement: qzaf039_Supplementary_Data [file qzaf039_supplementary_data.zip › Figure S4.pdf]

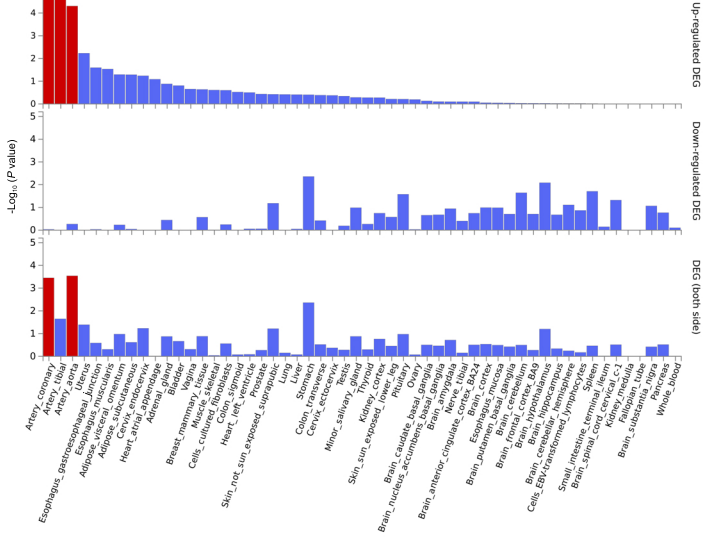

Supplement: qzaf039_Supplementary_Data [file qzaf039_supplementary_data.zip › Figure S5.pdf]

**A**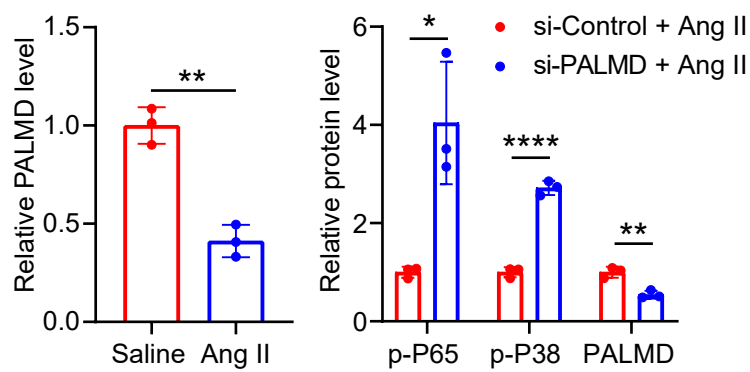**B**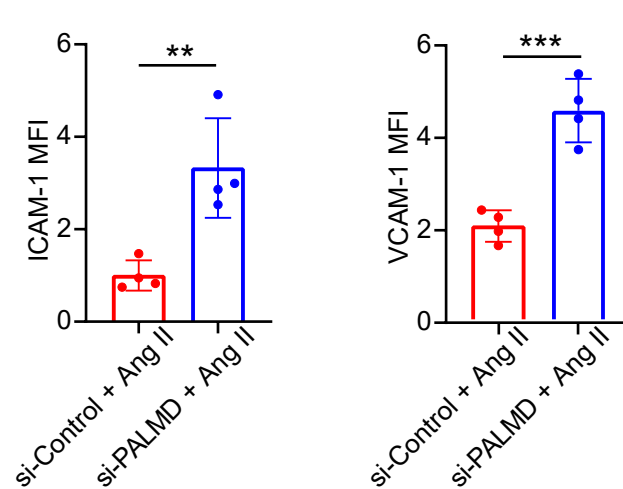**C**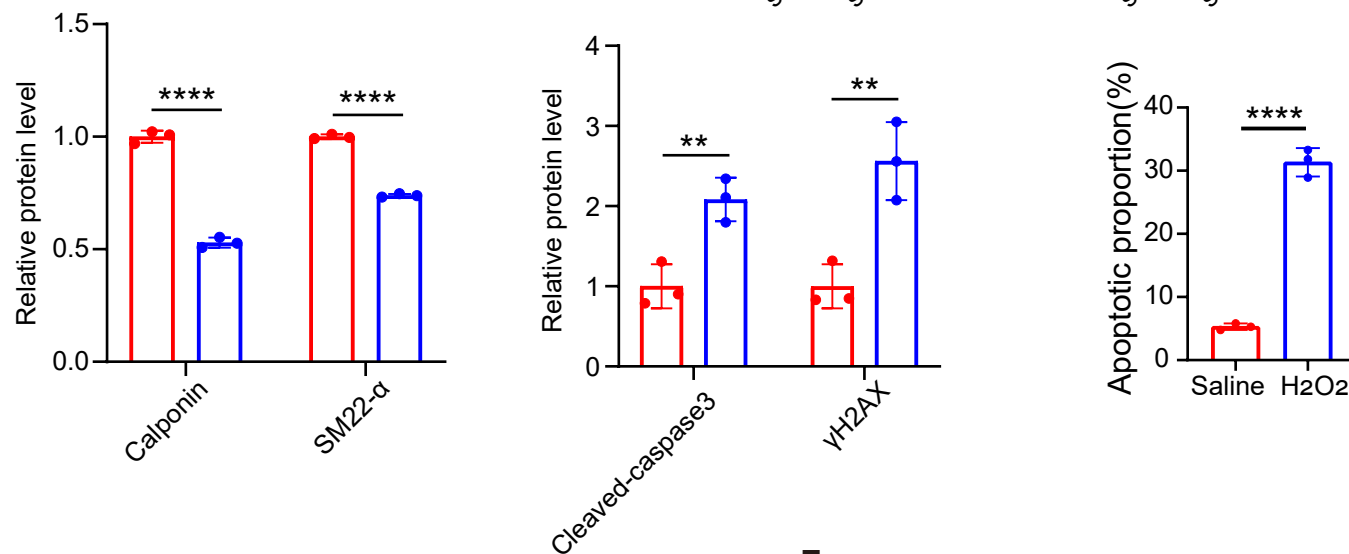**D**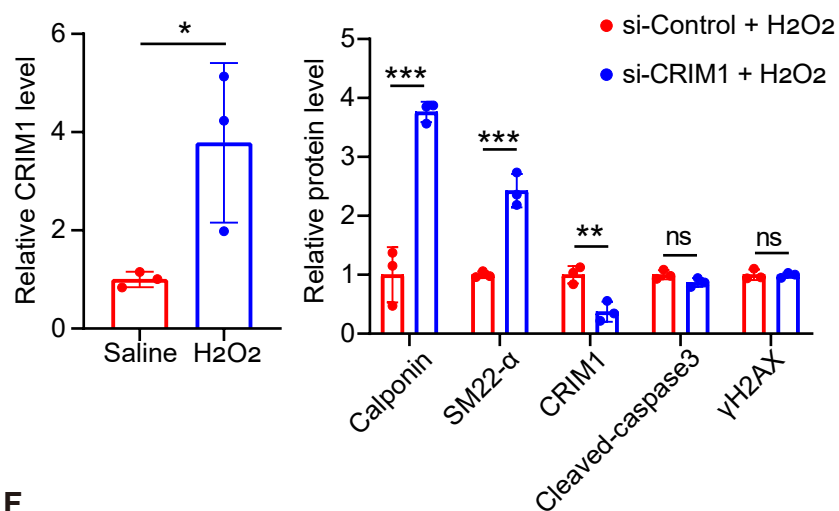**E**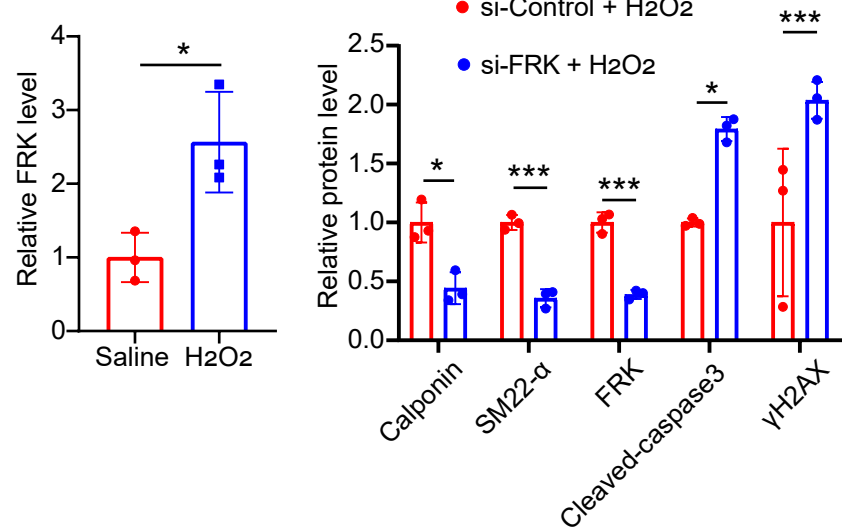**F**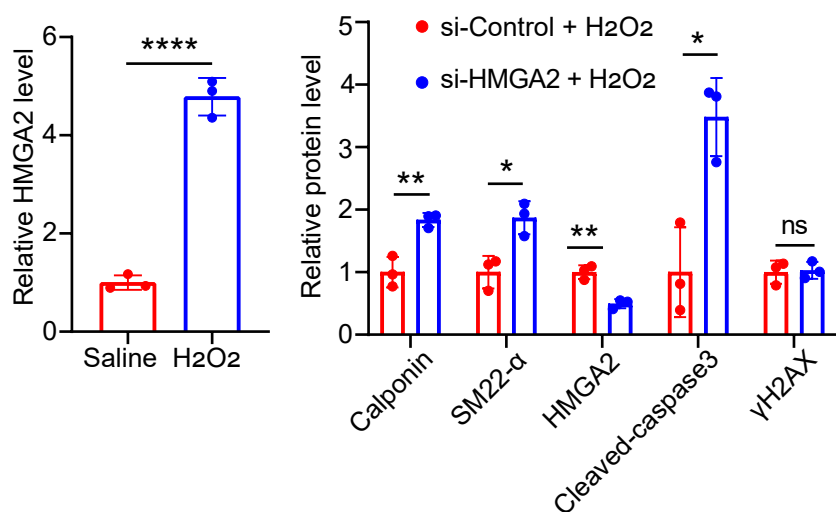**G**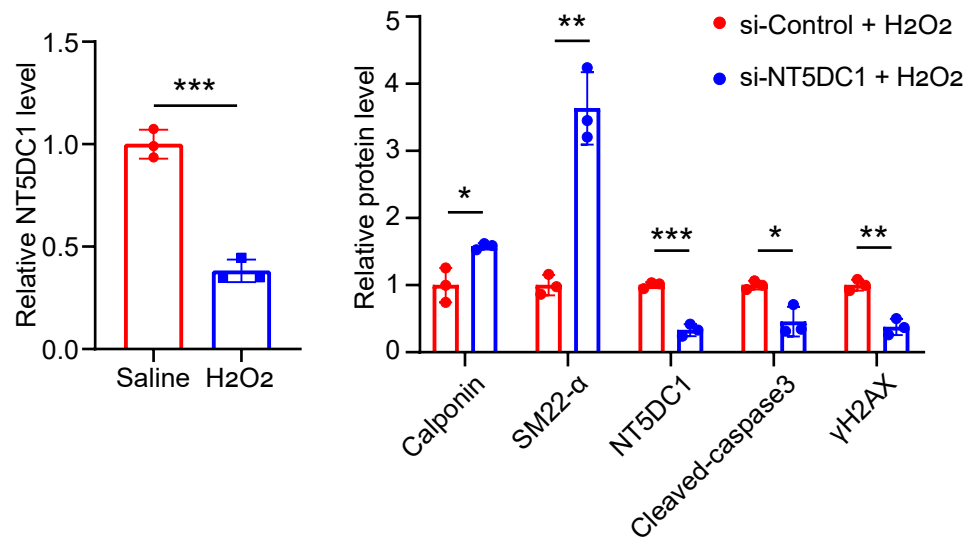**H**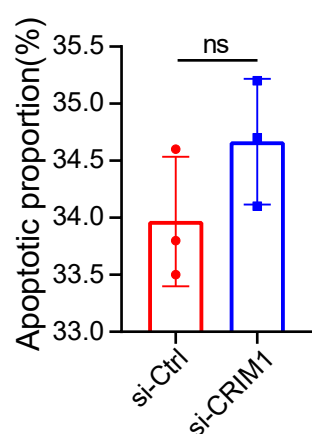**I**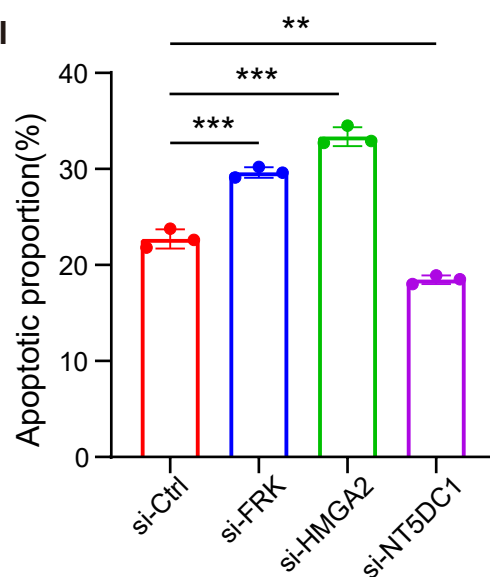

Supplement: qzaf039_Supplementary_Data [file qzaf039_supplementary_data.zip › Figure S6.pdf]

## Genetic correlation with AAD

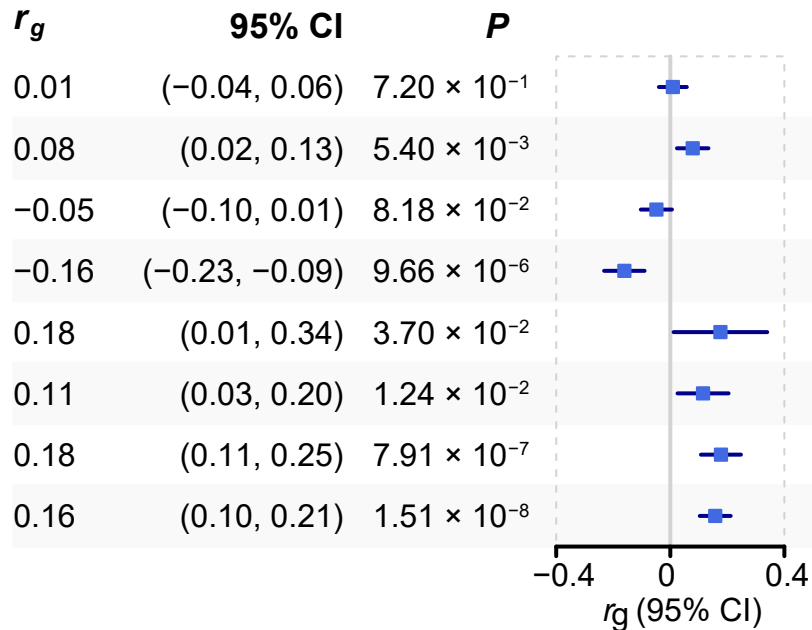

## MR: risk factors (exposure)–AAD (outcome)

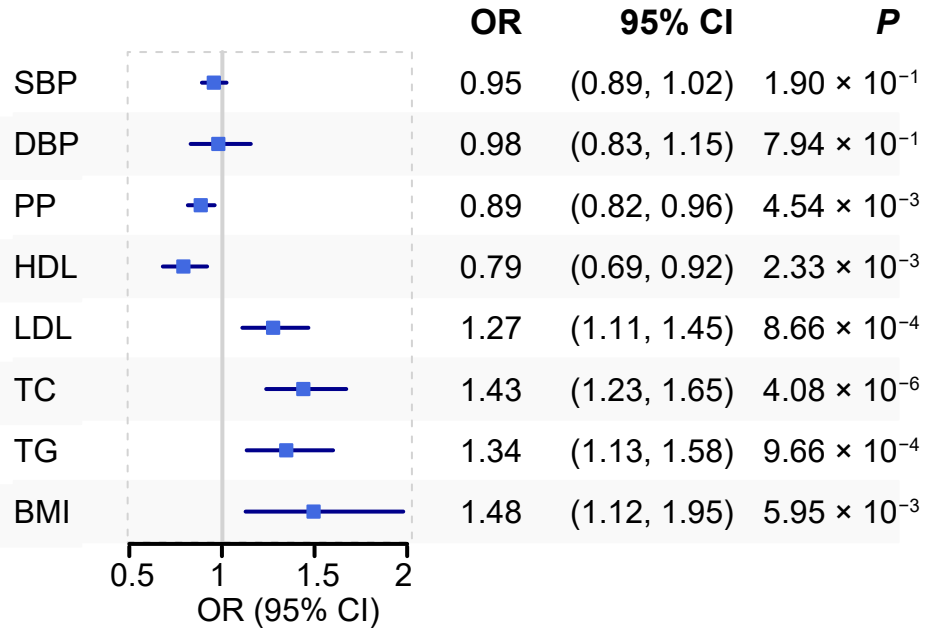

Supplement: qzaf039_Supplementary_Data [file qzaf039_supplementary_data.zip › Figure S7.pdf]

# MR: risk factors (exposure)–AAD (outcome)

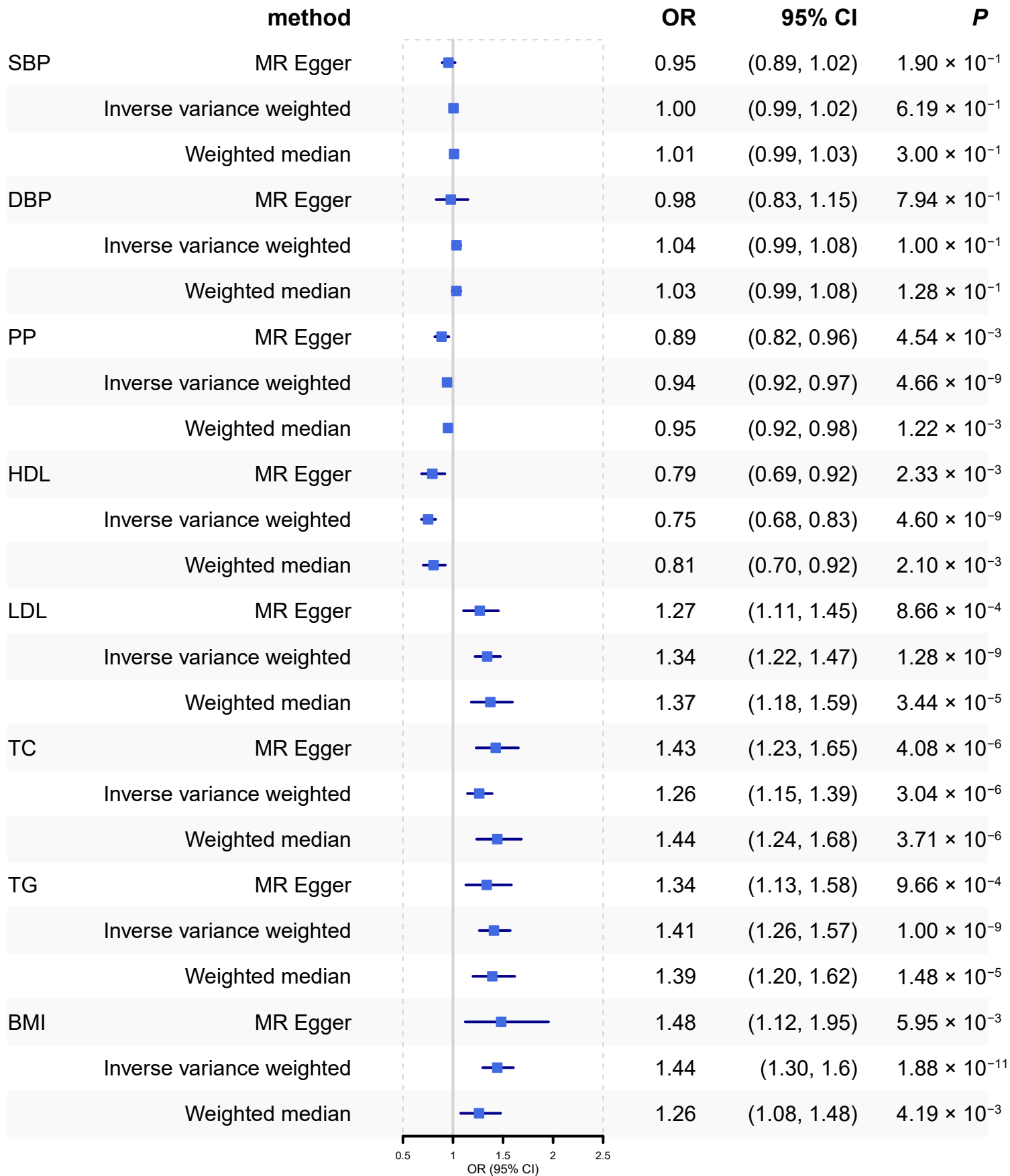

Supplement: qzaf039_Supplementary_Data [file qzaf039_supplementary_data.zip › Figure S8.pdf]
